# Acupuncture combined with biofeedback electrical stimulation for female stress urinary incontinence: a systematic review and meta-analysis

**DOI:** 10.3389/fmed.2026.1760125

**Published:** 2026-01-22

**Authors:** Hongji Liu, Aizizha Aikebai, Qianer Yuan, Jie Chen

**Affiliations:** Department of Acupuncture and Moxibustion, Dongguan Hospital of Traditional Chinese Medicine, Guangzhou University of Chinese Medicine, Dongguan, China

**Keywords:** acupuncture, biofeedback electrical stimulation, female, meta-analysis, stress urinary incontinence

## Abstract

**Introduction:**

This meta-analysis aimed to determine the clinical efficacy of acupuncture combined with biofeedback electrical stimulation for stress urinary incontinence in women.

**Methods:**

Databases including CNKI, WanFang, VIP, PubMed, Cochrane Library, Embase, and Web of Science were searched to collect randomized controlled trials (RCTs) on acupuncture combined with biofeedback electrical stimulation for female stress urinary incontinence from database inception to June 1, 2025, and performed a meta-analysis using Stata 15 software.

**Results:**

A total of 33 studies involving 2,860 patients were included in the analysis. Meta-analysis revealed that compared with the biofeedback electrical stimulation group, the acupuncture plus biofeedback electrical stimulation group significantly reduced the number of leakage episodes [SMD = −2.26, 95% CI (−3.42, −1.11)] and urine leakage volume [SMD = −1.79, 95% CI (−2.22, −1.37)], and ICIQ-SF scores [MD = −2.00, 95% CI (−2.61, −1.39)]. Additionally, the acupuncture plus biofeedback electrical stimulation group demonstrated significantly greater increases in pelvic floor muscle strength scores [SMD = 0.99, 95% CI (0.32, 1.65)]. The clinical efficacy of the acupuncture plus biofeedback electrical stimulation group was significantly higher than that of the biofeedback electrical stimulation control group [RR = 1.20, 95% CI (1.16, 1.25)].

**Conclusion:**

Acupuncture combined with biofeedback electrical stimulation therapy may offer certain advantages in treating female stress urinary incontinence. It may reduce the frequency and volume of urinary leakage, lower ICIQ-SF scores, increase pelvic floor muscle strength scores, and improve quality of life in women.

## 1 Introduction

Stress urinary incontinence (SUI) poses a significant threat to women’s health worldwide, is characterized by an involuntary loss of urine due to an increase in intra-abdominal pressure on exertion (e.g., exercising or lifting heavy objects), sneezing or coughing (1). Epidemiological data indicate that 10–40% of women worldwide suffer from SUI (2), with prevalence rates reaching 30–50% among women aged 45–59 (3). SUI is a chronic condition. Patients often avoid social interactions due to body odor caused by leakage, significantly impacting mental health and quality of life (4). Prolonged leakage can also lead to incontinence-associated dermatitis (5) and recurrent urinary tract infections (6), imposing heavy personal, familial, and socioeconomic burdens. In the United States, expenditures related to female urinary incontinence were estimated at over $206 million, rising to $246 million by 2013 (7).

Pathophysiologically, the recognized mechanisms of SUI primarily include urethral sphincter dysfunction and urethral hypermobility. Urethral sphincter dysfunction-type SUI mainly results from structural and functional impairment of the urethral sphincter due to peripheral or central neurological disorders, congenital developmental abnormality, chronic diseases, or aging (8). High urethral mobility-type SUI primarily results from weakened pelvic floor support structures, leading to insufficient support beneath the urethra and excessive downward displacement of the urethra during increased abdominal pressure (9). With aging, both the female urethral sphincter and its surrounding nerves undergo changes that diminish the function of pelvic floor support. Additionally, pregnancy or childbirth can cause relaxation of the urethral sphincter and nerve damage, resulting in urinary leakage during increased abdominal pressure. Therefore, promoting the recovery of the urethral sphincter and nerve function has become an effective clinical approach for treating female SUI. Clinical management of SUI patients is divided into surgical and non-surgical treatments. Surgical intervention carries certain risks and is not suitable for patients with mild to moderate SUI. Consequently, non-surgical treatments form the primary clinical approach, including electrical stimulation, pelvic floor muscle exercises, pelvic floor rehabilitation devices, and lifestyle interventions. Among these, electrical stimulation has emerged as a significant technique for treating pelvic floor dysfunction in recent years. It utilizes electrical currents to depolarize nerve cells, inhibit afferent nerve excitation, and promote urethral sphincter contraction, thereby suppressing involuntary urinary leakage (10). However, SUI exhibits a high recurrence rate, and electrical stimulation alone often fails to achieve satisfactory outcomes.

Acupuncture therapy constitutes one of the external treatment modalities within traditional Chinese medicine. Its techniques encompass conventional needle insertion, electroacupuncture, moxibustion with moxa cones, and moxibustion with indirect heat application. As a non-pharmacological, complementary intervention, it is characterized by its simplicity of application, safety, efficacy, and minimal adverse reactions (11). Its efficacy in adjunctive treatment for SUI has been extensively documented in research, yet high-quality evidence-based medical support remains insufficient. Published meta-analyses exhibit certain limitations (including inadequate study numbers and incomplete database coverage), with no studies conducting subgroup analyses for postpartum versus primary SUI. Furthermore, meta-analyses examining acupuncture combined with biofeedback for SUI treatment are lacking. Therefore, this study conducted a systematic search, collation, and selection of randomized controlled trials on acupuncture combined with biofeedback for SUI to perform a meta-analysis, aiming to provide evidence-based guidance for clinical decision-making.

## 2 Materials and methods

The systematic review was accepted through the U. S. National Institutes of Health’s online PROSPER International Prospective Registration System (CRD420251076360) (12).

### 2.1 Database search

Two trained researchers systematically searched China National Knowledge Infrastructure (CNKI), Wanfang, VIP, PubMed, Web of Science, Cochrane Library, and Embase from their inception to June 1, 2025, and the search has already captured the latest evidence before submission. The search strategy combined medical subject headings and free-text terms using the following keywords: (“acupuncture” or “warm needle acupuncture” or “electroacupuncture” or “moxibustion” or “auricular acupuncture” or “floating needle therapy” or “abdominal acupuncture” or “fire needle therapy”) AND (“biofeedback electrical stimulation” or “biofeedback”) AND (“stress urinary incontinence”). Detailed retrieval methods are provided in Supplementary material 1.

### 2.2 Inclusion criteria

(1) Study Type: Randomized controlled trials in Chinese or English. (2) Study Population: Female patients meeting diagnostic criteria for stress urinary incontinence (13), with no restrictions on etiology, disease duration, or age. (3) Intervention measures: In the original literature, the control group received biofeedback therapy or concurrent pelvic floor muscle training. The observation group supplemented the control group’s regimen with acupuncture treatment, comprising two traditional Chinese external therapies: acupuncture or moxibustion. Acupuncture modalities included simple needle insertion, electroacupuncture, warm needle moxibustion, moxibustion, indirect moxibustion, retention needles, and floating needles. (4) Outcome Measures: Frequency of leakage, volume of leakage, International Consultation on Incontinence Questionnaire Urinary Incontinence Short Form (ICI-Q-SF) score, and pelvic floor muscle strength score.

### 2.3 Exclusion criteria

(1) Duplicate publications; (2) Interventions in the control or observation group inconsistent with this study; (3) Non-clinical randomized controlled trials, such as reviews, case reports, conference proceedings, experiential reports, or animal studies; (4) Patients with stress urinary incontinence caused by other conditions such as medication, trauma, tumor compression, or other factors; (5) Primary literature with unclear reporting, poor quality, data errors or omissions, and lacking inclusion/exclusion criteria or efficacy standards.

### 2.4 Literature screening and data extraction

Initial and secondary screening of the literature was conducted using EndNote X9 software. Two researchers reviewed the titles and abstracts of all retrieved articles, excluding those clearly ineligible for inclusion. Full-text articles that potentially met the inclusion criteria underwent further screening to confirm eligibility. During the screening process, disputed articles were jointly discussed to determine whether they should be included or excluded. If consensus remained unresolved after two discussions, a third party was consulted. Data extraction from eligible studies was performed by two researchers, capturing the following: authors, publication year, title, sample size, mean age, primary disease type, intervention measures in the observation group, intervention measures in the control group, outcome measures, treatment frequency, and intervention cycle.

### 2.5 Quality assessment

Researchers L. H. J. and A. A. employed the Cochrane ROB 2.0 risk of bias assessment tool to evaluate the quality of the included studies (14). The assessment comprised five domains: randomization process, deviations of intended interventions, missing outcome data, measurement of the outcome, and selection of the reported result. Quality assessment criteria were categorized into three levels: low risk of bias, high risk of bias, and uncertain risk of bias. The two researchers conducted separate quality assessments of the included studies. In cases of disagreement, they resolved the matter through joint discussion or by consulting a third assessor.

### 2.6 Data analysis

Data from this study were statistically analyzed using Stata 15.0 software (Stata Corp, College Station, TX, United States). Continuous random variables—incontinence episodes, leakage volume, ICI-Q-SF scores, and pelvic floor muscle strength scores—were expressed as mean difference (MD), standardized mean difference (SMD) and 95% confidence intervals (CI). The overall response rate, being a dichotomous variable, was described as the relative risk (RR) with its 95% CI. *I*^2^ was used to assess the magnitude of heterogeneity. When *I*^2^ < 50%, a fixed-effect model was employed; if *I*^2^ ≥ 50%, indicating substantial heterogeneity, a random-effects model or subgroup analysis was used. Descriptive analysis was applied when heterogeneity testing revealed excessive heterogeneity in the data. Additionally, this study assessed publication bias through funnel plot analysis combined with the quantitative Egger’s test. If publication bias was detected, trim-and-trim methods were applied to evaluate its impact on study outcomes.

## 3 Results

### 3.1 Literature search results

**Figure 1:**
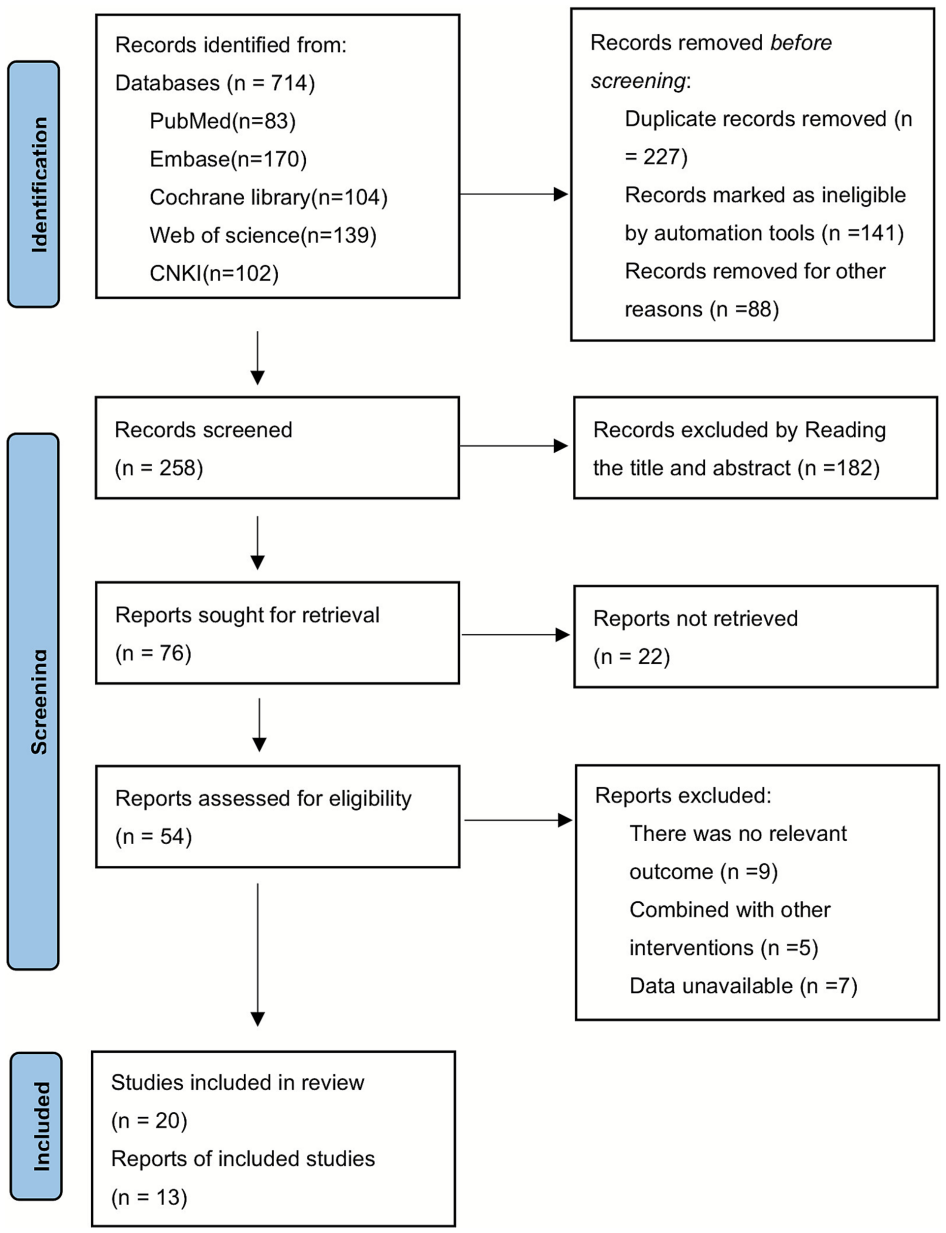
Literature search flow chart.

Two researchers conducted initial searches in Chinese and English databases, identifying a total of 714 relevant publications. After removing 456 duplicate records, they excluded 182 articles based on a screening of titles and abstracts. Additionally, 22 full-text articles were excluded due to unavailability. Following a thorough review of the full texts, 21 papers were ultimately removed from consideration, leaving 33 publications (15–47) for analysis, as illustrated in Figure 1.

### 3.2 Basic characteristics of included literature

Thirty-three research papers were included, comprising 20 clinical studies targeting pregnant women, 11 clinical studies targeting individuals with primary urinary incontinence, one clinical study targeting perimenopausal women, and one clinical study targeting postmenopausal women. The mean age in the acupuncture combined with biofeedback observation group was 40.26 ± 12.06 years, while the mean age in the biofeedback control group was 39.92 ± 11.98 years. Regarding intervention methods, among the 33 studies: 8 employed moxibustion (MOX), 8 employed acupuncture alone (AC), 7 utilized electroacupuncture (EA), 3 applied warm needle moxibustion (WNM), 2 combined acupuncture with electroacupuncture (AC + EA), 2 employed moxibustion with a barrier (MT), 1 combined acupuncture with warm needle moxibustion (AC + WNM), and 1 integrated traditional Chinese medicine with pressure acupuncture (TCM + PA) (See Table 1).

**Table 1:** Table of basic characteristics.

Study	Year	Sample size		Primary disease	Mean age (years)		Intervention		Outcomes
		EG	CG		EG	CG	EG	CG	
Li (24)	2024	45	45	Maternity	48.51	48.32	BES + WNM (Guanyuan, Qihai, Zhongji, Sanyinjiao, Mingmen, Zusanli, Shenshu, and Bladeshu) 30 min, twice/week; 4 weeks.	BES, 30 min, 2–3times/week, 12times	A1; A2;
Li et al. (15)	2017	60	60	Maternity	46.17	44.60	BES + MOX (Qihai, Guanyuan, Zhongji), 20 min, once/2 days, 10 times	BES,20 min, once/3 days, 10 times	A3; A4
Li et al. (16)	2022	49	47	Primary	50.00	51.00	BES + EA + AC (Acupuncture on the head combined with the “Sacro-Sepia Four Needling” therapy) 30 min, 3times/week, 4 weeks	BES, 20 min, 3times/week, 4 weeks	A3;
Deng (17)	2025	44	44	Maternity	29.15	29.12	BES + AC (Zusanli, Shenshu, Sanyingjiao), + WNM(Zhongji, Qihai), 30 min, 3times/week, 8 weeks	BES, 30 min, 2times/week, 8 weeks	A3
Fei et al. (18)	2020	32	30	Primary	53.67	52.42	BES + EA + AC (Acupuncture on the head combined with the Spleen Four Acupuncture Therapy), 30 min, 3times/week, 4 weeks	BES, 30 min, 3times/week, 4 weeks	A2; A3
Feng et al. (47)	2022	50	50	Maternity	32.19	32.16	BES + MOX (Perineal thunder fire moxibustion), 20 min, 2–3times/week, 20times	BES, 30 min, 2–3times/week, 20times	A2; A4
Hong et al. (19)	2023	30	30	Primary	53.9	50.4	BES + AC (Huiyang, bilateral Zhongliao), 30 min, once/2 days,10times	BES, 30 min, once/2 days, 10times	A2; A3; A4
Huang et al. (20)	2024	40	40	Primary	44.06	44.18	BES + AC + TCM (Guanyuan, Qihai, San YinJiao, Zusanli) AC:30 min, once/day; TCM:3times/day,1 month	BES, 20 min-25 min, once/day, 1 month	A1; A3; A4; A5
Huang (21)	2021	38	38	Primary	70.3	70.3	BES + MOX (Guanyuan, Qihai, Zhongji), 5 ~ 7 min,3times/week, 15times	BES, 30 min, 3times/week, 15 times	A1; A2; A4
Li and Liu (22)	2020	50	50	Maternity	27.73	27.98	BES + AC (Guanyuan, Zhongji, Shenshu, Bladder Meridian). 30 min, once/day, 30times	BES, 20 min, once/day, 30times	A4; A5
Li (23)	2021	39	39	Maternity	27.85	28.05	BES + AC (Szigong, Qihai, Shiquze, Taixi, Baiweishu, Baihui, Guanyuan, Taiyuan) + WNM(Sanyinjiao, Zusanli, Zhongji, Qihai), 30 min, 5times/week,8 weeks	BES, 30 min, 3-5times/week, 8 weeks	A4;
Li (25)	2016	50	50	Maternity	28.14	28.14	BES + AC (Guanyuan Qihai Zhongji, Zusanli) + MOX(guanyuan), 30 min,once/day,10times	BES, 20 min, twice/week, 5 weeks	A1; A2; A4
Liu et al. (26)	2024	132	132	Maternity	30.20	29.55	BES + WNM (Baihui, Zhongji, Sanyingjiao, Zhi Gong, Qihai, Zusanli, Taixi, Shizhe, Guan Yuan, Taiyan), 30 min, 3times/ week, 8 weeks	BES, 15–20 min, once/week, 8 weeks	A2; A4
Lu (27)	2018	60	60	Primary	49.8	48.5	BES + EA (“Sacral Four Points”), 50 min, twice/week, 20times	BES, 30 min, twice/week, 20times	A4
Ma (28)	2023	60	60	Maternity	36.12	35.94	BES + MOX (Perineum, Guanyuan, Qihai, San Yin Jiao), once/2 days, 20times	BES, 15 min, once/2 days, 20times	A2; A4;
Shu et al. (29)	2023	25	25	Primary	50.55	50.32	BES + EA (Zhongliao, Huiyang), 30 min, 3times/week,5 weeks	BES, 30 min, 3times/week, 5 weeks	A4
Tao et al. (30)	2024	55	55	Maternity	31.49	30.72	BES + MT (Shenque, Guanyuan, Shenshu, Zusanli, Sanyinjiao), 3times/week,4 weeks	BES, 30 min,3 times/week, 4 weeks	A2; A3; A4; A5;
Tian et al. (31)	2019	64	64	Perimenopause	50.4	50.4	BES + AC (Guanyuan, Qihai, Zhongji, Shizhui, Taixi, Sanyingjiao, Zusanli), 30 min,once/day,3 months	BES, 30 min, twice/week, 3 months	A3; A4
Tong et al. (32)	2017	19	19	Maternity	26.11	27.11	BES + EA (Guanyuan, Zhongji), 30 min, twice/week,5 weeks	BES, 30 min, twice/week, 5 weeks	A3
Wang et al. (33)	2018	20	20	Maternity	44.75	45.25	BES + MOX (Baihui, Guanyuan, Qihai, Zhongj) 15 min, once/day, 6 days, 4 weeks	BES, 30 min, once/2 days, 4 weeks	A3; A4
Wei (34)	2025	25	25	Maternity	36.11	32.52	BES + MOX (Shenque, Guanyuan, Zhongji, Zusanli, Pevac) 40 min, once/2 days,10times	BES, 30 min, once/2 days, 10times	A1; A2; A4
Wen et al. (35)	2018	30	30	Maternity	31.52	30.82	BES + WNM (ZuSanLi both on the body), ZhongJi, GuanYuan, 30 min, 10times	BES, 20 min, 2–3times/week, 20 min, 10times	A1; A2; A4
Xiang et al. (36)	2022	32	32	Maternity	29	29.3	BES + AC(Baihui, Guanyuan, Zusanli, Xiuyinjiao), 30 min, once/day, 5times. Six courses of mild treatment and eight courses of moderate treatment were given	BES,30 min, once/day,10times, There were 2 courses of mild treatment and 4 courses of moderate treatment	A2; A5
Yang et al. (37)	2018	32	33	Postmenopausal	55.1	55.1	BES + AC(Zhongliao, Sacral Four Points, Zhongji, Zhongwan, Guanyuan, Sanyingjiao), 25 min, 5times/week, 4 weeks	BES, 20 min, twice/week, 4 weeks	A1; A2; A3
Yuan et al. (38)	2024	30	30	Primary	53.27	52.52	BES + EA (Sacral Four Acupuncture Points), 30 min,once/day,20 days	BES, 30 min, once/day, 20 days	A2; A3; A4
Zhang et al. (39)	2025	55	55	Maternity	28.49	28.51	BES + EA (Sacral Four Acupuncture Points), 20 min, twice/week, 5 weeks	BES, 15 min, once/3 days, 20times	A4
Zheng et al. (40)	2022	49	49	Maternity	61.98	62.51	BES + EA (Zhongji, Qugu, Guilai, Qi chong, Bilateral Zhongliao, Ciliao, Shenshu, Huiyang) + AC(Panguangshu Jingmen, Shuidao, Qihai, Guanyuan, Guilai),30 min,5times/week,12 weeks	BES, 20 min, twice/week, 12 weeks	A3
Zheng et al. (41)	2020	30	30	Maternity	29.29	28.72	BES + MOX (Pishu, Qihai, Sanyinjiao, Shenshu, Zusanli, Guanyuan), 30–40 min, 2–3 times/week, 4 weeks	BES, 30 min, 2–3times/week, 4–5 weeks	A3; A4;
Zhou et al. (42)	2021	42	41	Maternity	28.83	27.16	BES + TCM + PA (Qi Hai, Zhongwan, Xiawan, Zhongji, Guanyuan) + MT (Shenshu, Pishu, Panguangshu), 20 min, once/2 days, 3times/week, 8 weeks	BES,30 min, once/2 days, 3times/week, 8 weeks	A2; A3; A4; A5
Zhou et al. (43)	2021	41	41	Maternity	26.9	27.2	BES + MT (Shenshu, Pishu, Panguangshu) + PA(Zhongwan, Xiawan, Zhongji, Qihai, Guanyuan), 20 min, once/2 days, 3times/week, 8 weeks	BES, 30 min, once/2 days, 3times/week, 8 weeks	A2; A3; A4; A5
Zhou et al. (44)	2023	30	30	Primary	nr	nr	BES + MOX (Long Snake Moxibustion), 30 min,3times/week,8 weeks	BES, 30 min, 3times/week, 8 weeks	A3; A4
Tian et al. (45)	2024	30	30	Primary	40.47	40.57	BES + AC (Guanyuan, Qihai, Zhongji, Zusanli, Sanyinjiao,and Yinlingquan) 30 min, 5times/week, 10times	BES, 30 min, 3times/week, 15times	A2; A3; A4
Xinmei et al. (46)	2024	44	44	Primary	36.2	38.2	BES + EA (“foursacralneedles”), 30 min, twice/week, 15times.	BES, twice/week, 15times.	A2; A4

EG, Observation group; CG, Control group; nr, not report; WNM, Warm needle moxibustion; BES, Biofeedback electrical stimulation; MOX, Moxibustion; EA, Electroacupuncture; AC, Acupuncture; TCM, Traditional Chinese medicine; PA, Press Acupuncture; MT, Moxibustion through an object; A1, The number of urine leakage; A2, Urine leakage volume; A3, ICI-Q-SF score; A4, Clinical efficacy; A5, Pelvic floor muscle strength.

### 3.3 Risk of bias assessment

**Figure 2:**
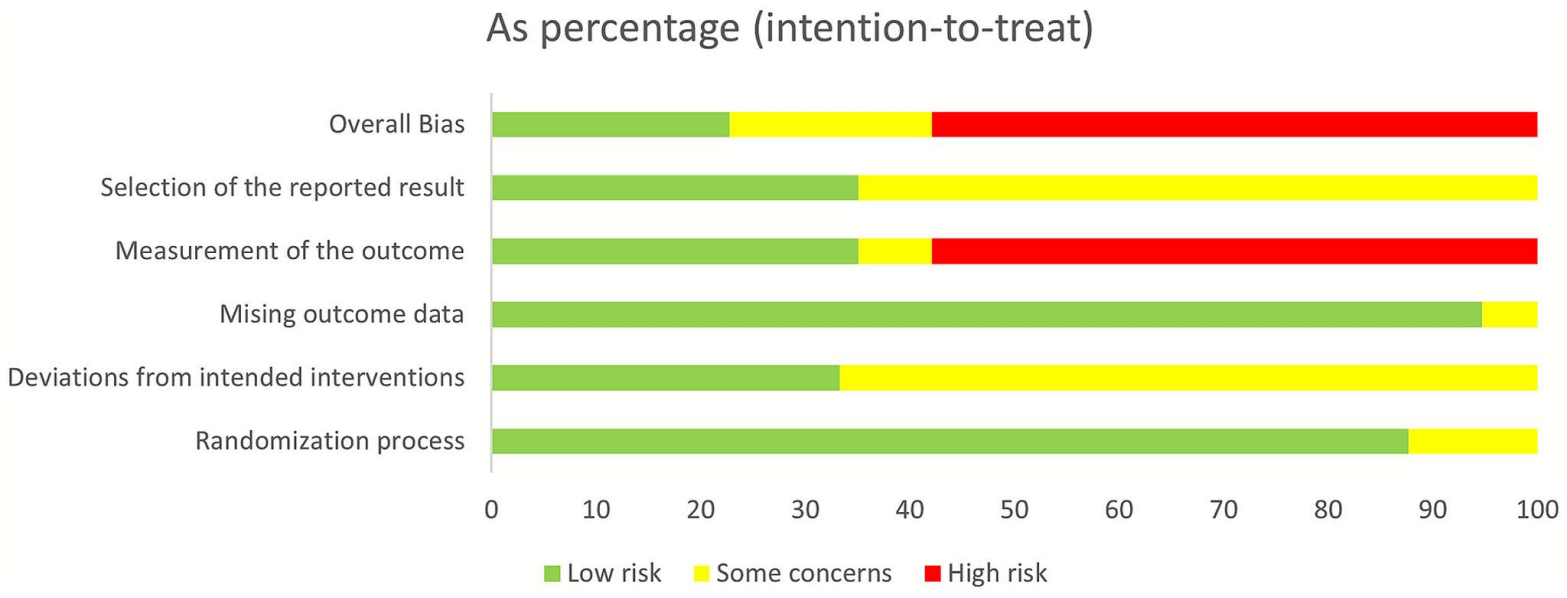
Risk bias of summary.

**Figure 3:**
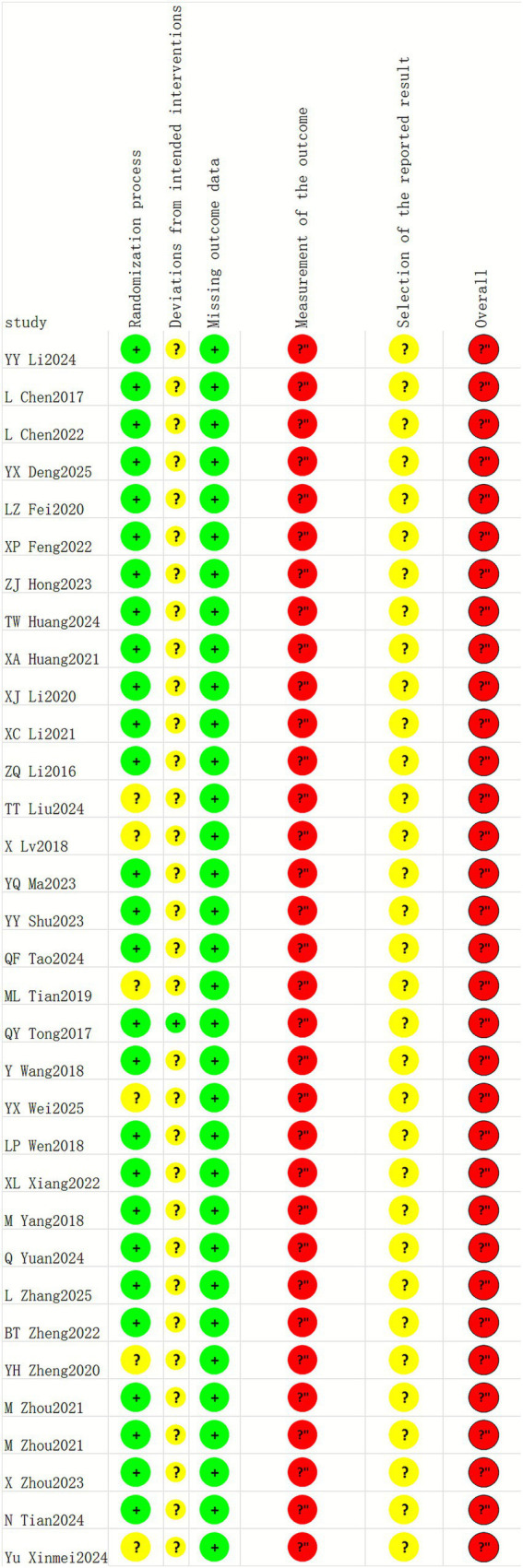
Risk bias of graph.

Among the included literature studies, 29 employed random sequence generation and were judged to be at low risk of bias; 7 studies did not specify the randomization method and were judged to be at uncertain risk. One study provided sufficient methodological detail regarding intervention deviation and was thus judged to be at low risk of bias. In contrast, 32 studies did not specify whether intervention deviation occurred and were judged to be at uncertain risk. No studies reported missing data; thus, all 33 studies were assessed as low risk for this outcome. Given the nature of acupuncture, blinding was difficult to achieve; consequently, all 33 studies were assessed as being at high risk for measurement of the outcome. The majority of included studies were in Chinese-language publications, and 33 studies were assessed as being at low risk for selection of the reported result. See Figures 2, 3.

### 3.4 Data analysis

#### 3.4.1 Clinical efficacy

**Figure 4:**
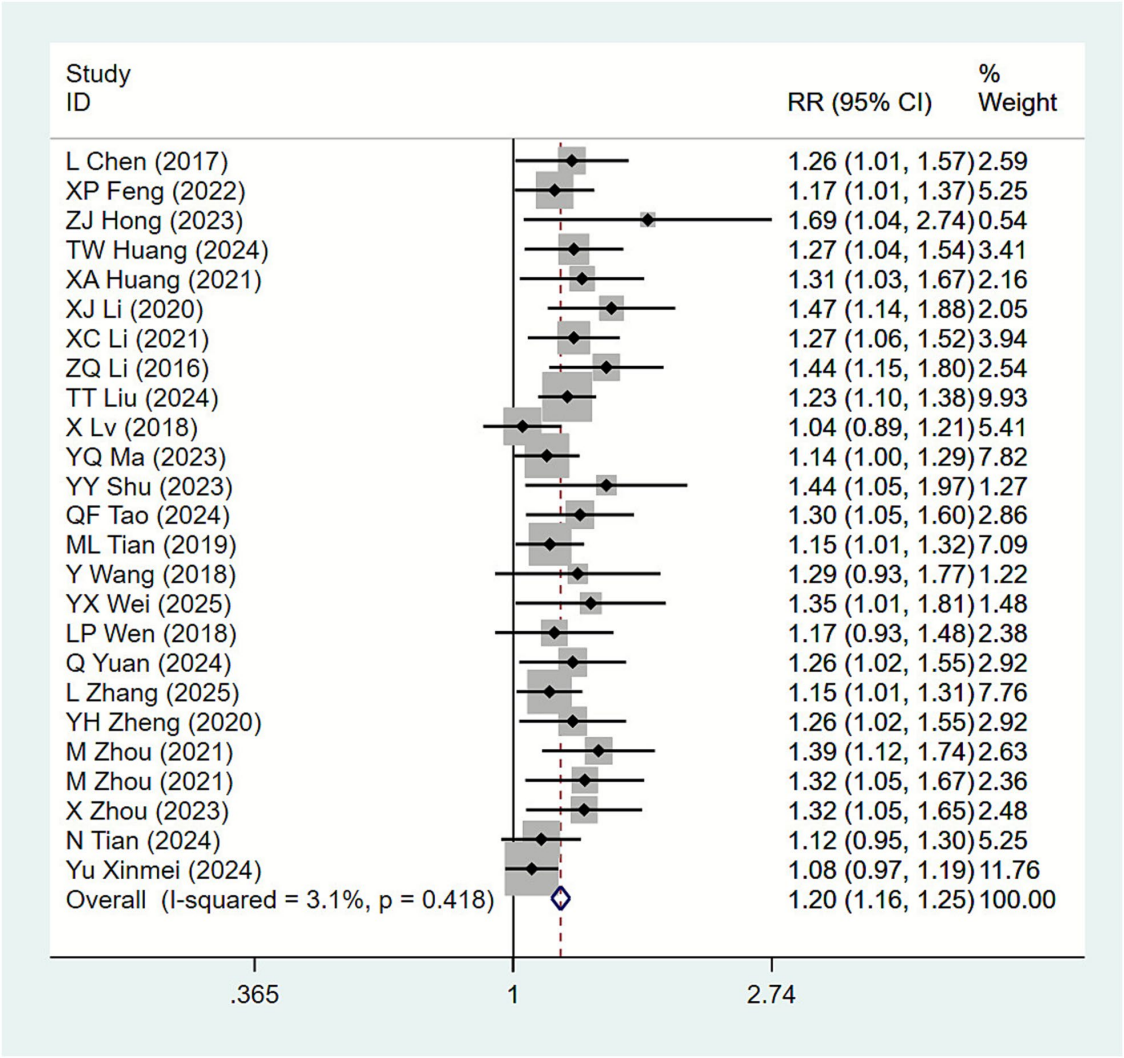
Forest plot of meta-analysis of clinical efficacy.

Among the 33 included studies, 25 (15, 19, 21–23, 25–31, 33–35, 38, 39, 41–47) reported clinical efficacy. Conduct heterogeneity tests for each study, *I*^2^ = 3.1%; thus, fixed-effect model analysis was applied. Results (Figure 4) indicate that, compared with the biofeedback electrical stimulation group, the acupuncture plus biofeedback electrical stimulation group showed a significantly improved clinical efficacy in SUI patients [RR = 1.20, 95% CI (1.16, 1.25)]. Subgroup analyses based on different acupuncture interventions revealed (Table 2) that MOX [RR = 1.22, 95% CI (1.14, 1.31)], AC [RR = 1.23, 95% CI (1.13, 1.34)], and EA [RR = 1.12, 95% CI (1.05, 1.20)] indicated superior clinical efficacy compared to the control group for treating female stress urinary incontinence. Subgroup analysis by primary disease type (Table 3) revealed that acupuncture combined with biofeedback demonstrated significant clinical efficacy for stress urinary incontinence in women across different primary disease categories: Maternity [RR = 1.24, 95% CI (1.18, 1.30)], Primary [RR = 1.16, 95% CI (1.09, 1.23)]. Subgroup analysis based on acupuncture treatment duration (Table 4) revealed that 20 days [RR = 1.30, 95% CI (1.15, 1.48)], 5 weeks [RR = 1.18, 95% CI (1.09, 1.27)], and 8 weeks [RR = 1.28, 95% CI (1.18, 1.38)], indicated that acupuncture combined with biofeedback demonstrated superior clinical efficacy compared to the control group.

**Table 2:** Subgroup analysis of different acupuncture interventions.

Outcome	Group	Subgroup	No of study	Heterogeneity		SMD(95%CI)
				*I*^2^ (%)	*p*	
Number of urine leakage	Type of interevntion	WNM	2	84.1	0.012	−0.91 (−1.77, −0.05)
		AC + TCM	1	0	0	−8.11 (−9.47, −6.74)
		MOX	2	0.0	0.519	−1.40 (−1.80, −1.01)
		AC	2	98.4	0.0001	−1.98 (−5.16, 1.21)
Urine leakage volume	Type of interevntion	WNM	3	96.7	0.0001	−1.26 (−2.58, 0.06)
		EA + AC	1	0	0	−1.15 (−1.69, −0.61)
		MOX	4	93.3	0.0001	−2.33 (−3.41, −1.24)
		AC	5	93.9	0.0001	−2,25 (−3.37, −1.12)
		EA	2	0	0.804	−0.98 (−1.32, −0.64)
		MT	1	0	0	−1.05 (−1.45, −0.65)
		TCM + PA + MT	1	0	0	−2.27 (−2.82,-1.71)
		MT + PA	1	0	0	−1.87 (−2.39, −1.34)
ICIQSF score	Type of interevntion	MOX	4	74.1	0.009	−2.18 (−3.41,-0.95)
		EA + AC	3	58.3	0.091	−1.37 (−2.08,-0.66)
		WNM + AC	1	0	0	−3.91 (−4.45,-3.37)
		AC	4	88.4	0.0001	−1.40 (−2.77,-0.03)
		AC + TCM	1	0	0	−1.21 (−1.81,-0.61)
		MT	1	0	0	−1.79 (−2.10,-1.48)
		EA	2	54.5	0.138	−1.14 (−2.21,-0.07)
		TCM + PA + MT	1	0	0	−3.72 (−4.19,-3.25)
		MT + PA	1	0	0	−3.60 (−4.08,-3.12)
Clinical efficacy	Type of interevntion	MOX	8	0.0	0.900	1.22 (1.14,1.31)
		AC	5	49.1	0.097	1.23 (1.13,1.34)
		AC + TCM	1	0	0	1.27 (1.04,1.54)
		AC + WNM	1	0	0	1.27 (1.06,1.52)
		WNM	2	0.0	0.700	1.22 (1.10,1.35)
		EA	5	26.0	0.248	1.12 (1.05,1.20)
		MT	1	0	0	1.30 (1.05,1.60)
		TCM + PA + MT	1	0	0	1.39 (1.12,1.74)
		MT + PA	1	0	0	1.32 (1.05,1.67)

**Table 3:** Subgroup analysis of primary disease type.

Outcome	Group	Subgroup	No of study	Heterogeneity		SMD (95% CI)
				*I*^2^ (%)	*p*	
Number of urine leakage	Type of primary disease	Maternity	4	94.8	0	−1.73 (−2.95, −0.52)
		Primary	2	98,8	0.0001	−4.67 (−11.34, 1.99)
		Postmenopausal	1	0	0	−0.36 (−0.85, 0.13)
Urine leakage volume	Type of primary disease	Maternity	11	92.9	0.0001	−1.86 (−2.40, −1.31)
		Primary	6	91.9	0.0001	−1.64 (−2.44, −0.83)
		Postmenopausal	1	0	0	−2.09 (−2.71, −1.48)
ICIQSF score	Type of primary disease	Maternity	9	96.3	0.0001	−2.48 (−3.39,-1.57)
		Primary	7	62.8	0.013	−1.22 (−1.82,-0.62)
		Perimenopause	1	0	0	−1.70 (−2.74,-0.66)
		Postmenopausal	1	0	0	−2.60 (−3.40,-1.80)
Clinical efficacy	Type of primary disease	Maternity	15	0.0	0.823	1.24 (1.18,1.30)
		Primary	9	33.6	0.149	1.16 (1.09,1.23)
		Perimenopause	1	0	0	1.15 (1.01,1.32)

**Table 4:** Subgroup analysis of treatment duration.

Outcome	Group	Subgroup	No of study	Heterogeneity		SMD (95% CI)
				*I*^2^ (%)	*p*	
Number of urine leakage	Type of treatment duration	4 weeks	2	87.8	0.004	−0.85 (−1.81, 0.11)
		1 month	1	0	0	−8.11 (−9.47, −6.74)
		5 weeks	2	81.2	0.021	−0.88 (−1.71, −0.06)
		10 days	1	0	0	−3.61 (−4.25, −2.96)
		20 days	1	0	0	−1.57 (−2.21,-0.93)
Urine leakage volume	Type of treatment duration	4 weeks	4	67.3	0.027	−1.29 (−1.72, −0.87)
		10 weeks	1	0	0	−2.16 (−2.66, −1.66)
		20 days	3	69.8	0.036	−1.18 (−1.79, −0.57)
		5 weeks	2	98.5	0.0001	−2,25 (−6.19, 1.69)
		10 days	1	0	0	−4.58 (−5.34, −3.82)
		8 weeks	3	41.5	0.181	−2.24 (−2.58, −1.90)
		40 days	1	0	0	−1.20 (−1.59,-0.81)
		5 days	1	0	0	−1.96 (−2.57, −1.36)
		2 weeks	1	0	0	−1.96 (−2.58,-1.34)
		7.5 weeks	1	0	0	−0.94 (−1.39,-0.50)
ICIQSF score	Type of treatment duration	20 days	3	52.3	0.123	−2.24 (−3.34,-1.15)
		4 weeks	5	39.6	0.142	−2.06 (−2.53,-1.59)
		8 weeks	4	87.9	0.0001	−3.20 (−4.03,-2.37)
		1 month	1	0	0	−1.21 (−1.81,-0.61)
		3 month	1	0	0	−1.70 (−2.74,-0.66)
		5 weeks	1	0	0	−0.74 (−1.36,-0.12)
		12 weeks	1	0	0	−0.93 (−1.22,-0.64)
		2 weeks	1	0	0	−0.00 (−0.65,0.65)
Clinical efficacy	Type of treatment duration	20 days	4	0.0	0.711	1.30 (1.15,1.48)
		10 weeks	2	20.8	0.261	1.10 (0.99,1.23)
		1 month	1	0	0	1.27 (1.04,1.54)
		5 weeks	5	0	0.625	1.18 (1.09,1.27)
		30 days	1	0	0	1.47 (1.14,1.88)
		8 weeks	5	0	0.890	1.28 (1.18,1.38)
		10 days	1	0	0	1.44 (1.15,1.80)
		4 weeks	3	0	0.982	1.28 (1.12,1.46)
		3 month	1	0	0	1.15 (1.01,1.32)
		2 weeks	1	0	0	1.12 (0.95,1.30)
		7.5 weeks	1	0	0	1.08 (0.97,1.19)

#### 3.4.2 Frequency of urinary incontinence episodes

**Figure 5:**
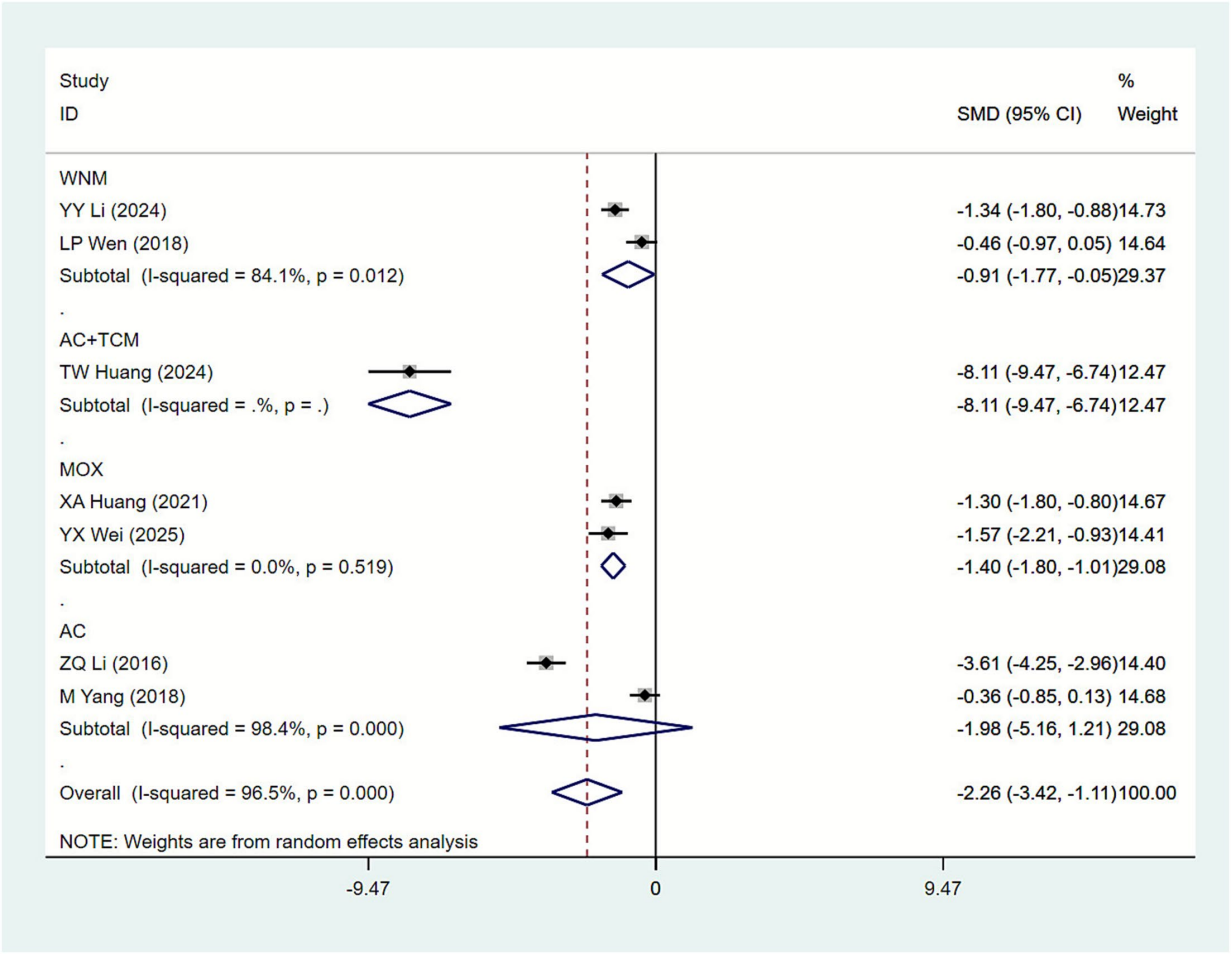
Forest plot of meta-analysis of frequency of urinary incontinence episodes.

Seven studies (20, 21, 24, 25, 34, 35, 37) reported urinary incontinence episode frequency as an outcome measure. The heterogeneity test in the study revealed: *I*^2^ = 96.5%. Therefore, data were analyzed using a random-effects model. Results (Figure 5) indicate that, compared with the biofeedback electrical stimulation group, the acupuncture plus biofeedback electrical stimulation group showed a significantly reduced number of urinary episodes in SUI patients [SMD = −2.26, 95% CI (−3.42, −1.11)]. Subgroup analysis by acupuncture intervention type (Table 2) revealed that WNM [SMD = −0.91, 95% CI (−1.77, −0.05)] and MOX [SMD = −1.40, 95% CI (−1.80, −1.01)] indicated that different interventions reduced the frequency of urinary leakage in women with stress urinary incontinence. Subgroup analysis by primary disease type (Table 3) showed: Maternity [SMD = −1.73, 95% CI (−2.95, −0.52)]. Subgroup analysis based on acupuncture treatment duration revealed the following results (Table 4): 5 weeks [SMD = −0.88, 95% CI (−1.71, −0.06)].

#### 3.4.3 Urinary leakage volume

**Figure 6:**
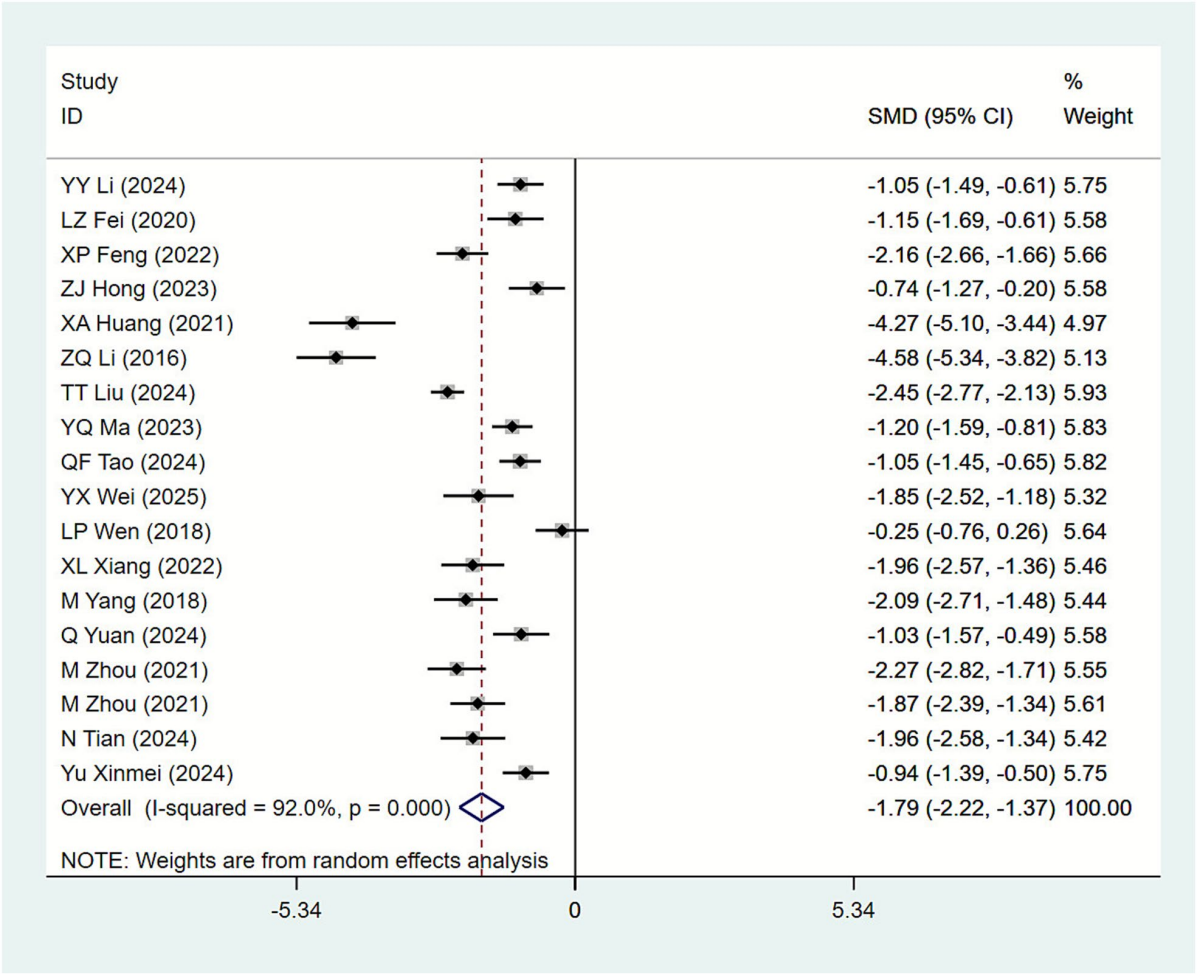
Forest plot of meta-analysis of urinary leakage volume.

Among the included studies, 18 publications (18, 19, 21, 24–26, 28, 30, 34–38, 42, 43, 45–47) reported urinary leakage volume as an outcome measure. The heterogeneity test in the study revealed I^2^ = 92.0%. Therefore, a random-effects model was used for meta-analysis. The heterogeneity test (Figure 6) indicated that, compared with the biofeedback electrical stimulation therapy group, the acupuncture combined with biofeedback electrical stimulation group showed a significantly reduced urinary leakage volume in SUI patients [SMD = −1.79, 95% CI (−2.22, −1.37)]. Subgroup analysis based on different acupuncture intervention measures showed (Table 2) that MOX [SMD = −2.33, 95% CI (−3.41, −1.24)], AC [SMD = −2.25, 95% CI (−3.37, −1.12)], and EA [SMD = −0.98, 95% CI (−1.32, −0.64)] all had significant effects in reducing urine leakage volume. Subgroup analysis by underlying disease type (Table 3) showed: Maternity [SMD = -1.86, 95%CI (−2.40, −1.31)], Primary[SMD = -1.64, 95%CI(−2.44, −0.83)]. The acupuncture combined with biofeedback electrical stimulation group demonstrated significant efficacy in improving urinary leakage volume across patients with different primary disease types. Subgroup analysis based on acupuncture treatment duration revealed the following results (Table 4): 4 weeks [SMD = −1.29, 95% CI (−1.72, −0.87)], 20 days [SMD = −1.18, 95% CI (−1.79, −0.57)], and 8 weeks [SMD = −2.24, 95% CI (−2.58, −1.90)]. These findings indicate that acupuncture combined with biofeedback therapy demonstrates significant therapeutic efficacy, with longer treatment durations yielding greater reductions in urinary leakage volume.

#### 3.4.4 ICI-Q-SF scores

**Figure 7:**
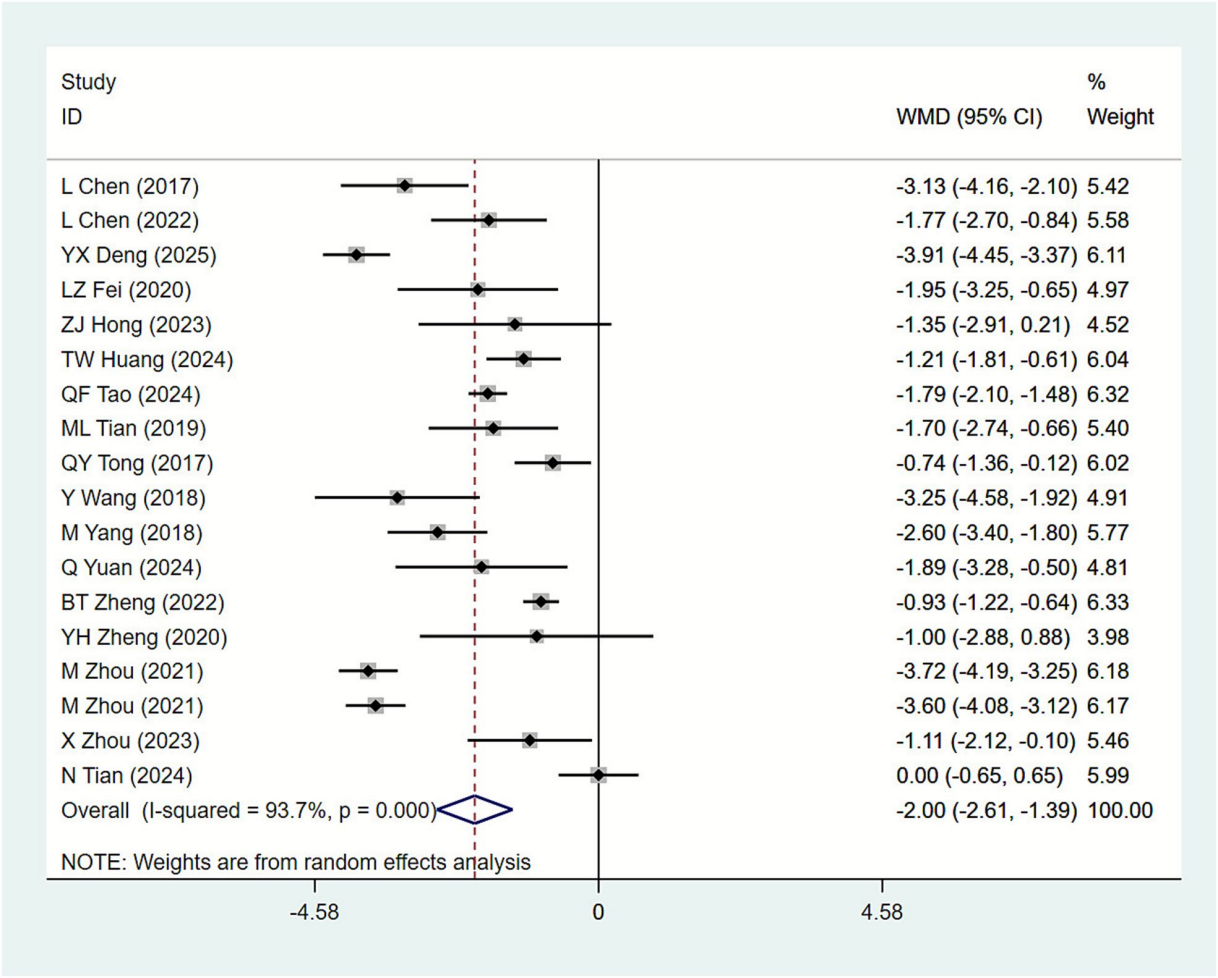
Forest plot of meta-analysis of ICI-Q-SF scores.

Eighteen studies (15–20, 30–33, 37, 38, 40–45) reported ICI-Q-SF scores, where lower scores indicate better treatment efficacy. Heterogeneity analysis *I*^2^ = 93.7% prompted the use of a random-effects model for meta-analysis. Results (Figure 7) show: Compared with biofeedback electrical stimulation electrical stimulation therapy alone, acupuncture combined with biofeedback electrical stimulation significantly reduced ICI-Q-SF scores in SUI patients [MD = −2.00, 95% CI (−2.61, −1.39)]. Subgroup analysis by acupuncture intervention type (Table 2) revealed: MOX [MD = −2.18, 95% CI (−3.41, −0.95)], EA + AC [MD = −1.37, 95% CI (−2.08, −0.66)], AC [MD = −1.40, 95% CI (−2.77, −0.03)], and EA [MD = −1.14, 95% CI (−2.21, −0.07)] all significantly reduced ICI-Q-SF scores. Subgroup analysis by primary disease type (Table 3) revealed: Maternity [MD = -2.48, 95%CI (−3.39,-1.57)], Primary[MD = -1.22, 95%CI(−1.82,-0.62)]. The acupuncture combined with biofeedback electrical stimulation group showed significant improvement in ICI-Q-SF scores across all primary disease types, with greater efficacy observed in patients with postpartum stress urinary incontinence. Subgroup analysis based on acupuncture treatment duration revealed (Table 4) that 20 days [MD = −2.24, 95% CI (−3.34, −1.15)], 4 weeks [MD = −2.06, 95% CI (−2.53, −1.59)], and 8 weeks [MD = −3.20, 95% CI (−4.03, −2.37)]. These findings indicate that acupuncture combined with biofeedback therapy demonstrates significant therapeutic efficacy, with greater reductions in ICI-Q-SF scores observed at longer treatment durations.

#### 3.4.5 Pelvic floor muscle strength scores

**Figure 8:**
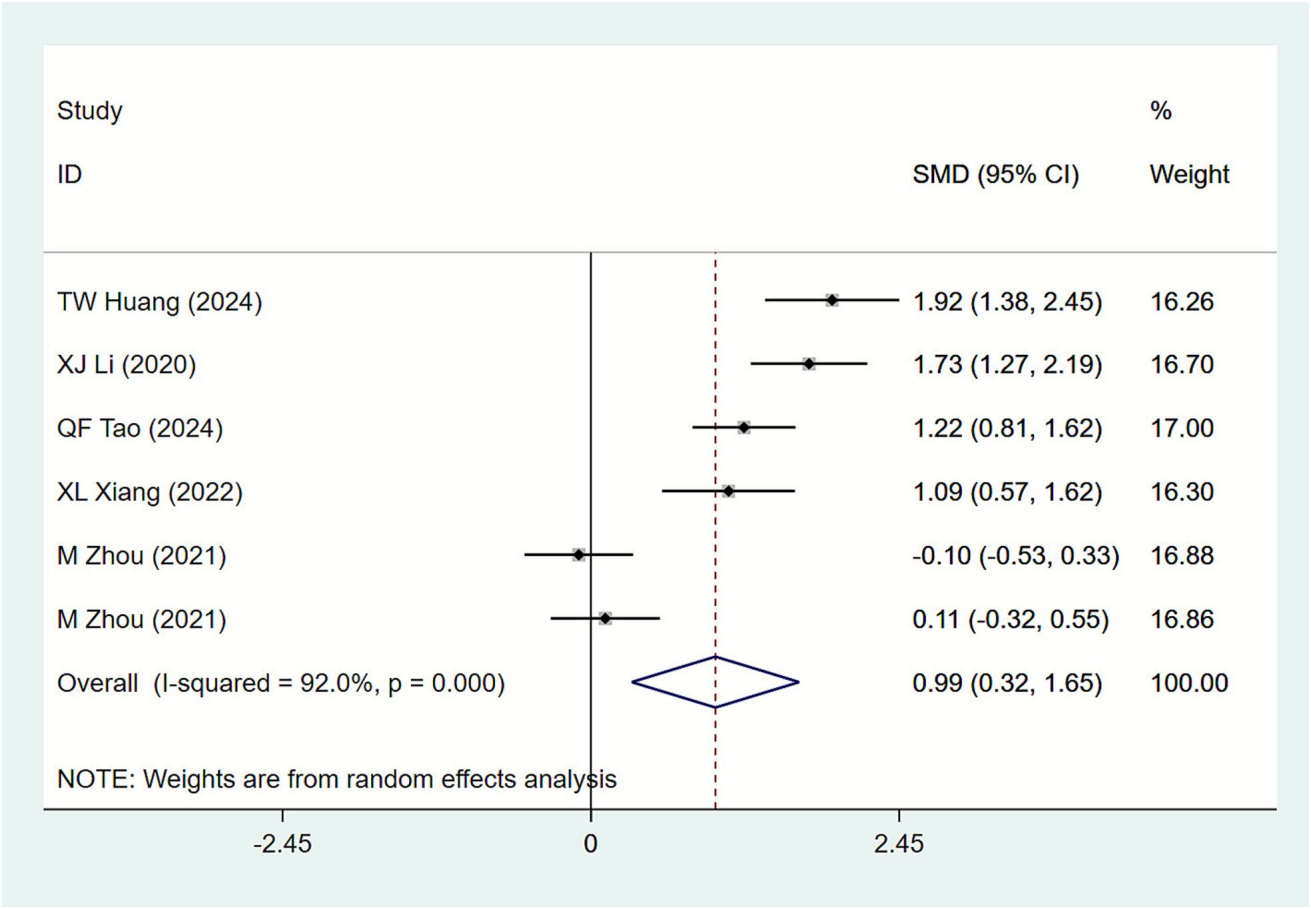
Forest plot of meta-analysis of pelvic floor muscle strength scores.

Six studies (20, 22, 30, 36, 42, 43) reported pelvic floor muscle strength scores. The heterogeneity test in the study showed that *I*^2^ = 92.0%. Therefore, a random effects model was adopted for the Meta-analysis. Results (Figure 8) indicate: Compared with biofeedback electrical stimulation therapy alone, acupuncture combined with biofeedback electrical stimulation significantly improved pelvic floor muscle strength scores in SUI patients [SMD = 0.99, 95% CI (0.32, 1.65)]. Subgroup analysis was not performed due to the limited number of studies.

### 3.5 Sensitivity analysis

Analysis of heterogeneity tests revealed significant heterogeneity in urinary leakage frequency, leakage volume, ICI-Q-SF scores, and pelvic floor muscle strength scores. Therefore, sensitivity analysis was conducted using a stepwise exclusion method on relevant data for the five included outcome measures. Results (Supplementary Figures 1–4) showed no significant changes in pooled effect sizes, indicating robust study findings.

### 3.6 Publication Bias

Funnel plots for urinary leakage frequency, ICI-Q-SF scores, and clinical efficacy (Supplementary Figures 5–7) exhibited uneven distributions on both sides. Egger tests were, respectively, *p* = 0.012, *p* = 0.042, and *p* = 0.000. Using the quantitative Egger test as the standard, this indicates that publication bias may be present in the results for these three outcome measures. Therefore, the trim-and-treat method (Supplementary Figure 8) was employed to assess the pooled results. Funnel plots for urinary leakage volume and pelvic floor muscle strength scores (Supplementary Figures 11, 12) showed relatively uniform bilateral distributions, with Egger tests yielding *p* = 0.107 and *p* = 0.314, respectively, indicating minimal publication bias.

## 4 Discussion

SUI falls under the category of “urinary incontinence” in Traditional Chinese Medicine (TCM), with a pathological location in the bladder and close relationships with the kidneys, spleen, and lungs. Its pathogenesis primarily manifests as kidney qi deficiency, leading to impaired retention, and spleen qi deficiency failing to regulate body fluids. Treatment focuses on tonifying the kidney to consolidate the foundation, strengthening the spleen to boost qi, and lifting and consolidating retention (48). From a modern medical perspective, SUI development is associated with urethral sphincter dysfunction and increased urethral activity, while normal neural regulation and maintenance of urethral pressure are equally critical (49). Acupuncture exerts its effects through neuroendocrine mechanisms, offering the unique advantage of non-pharmacological intervention. By stimulating neural control points of the bladder and sphincter, it induces needle sensations. Different needling techniques (stimulation patterns and intensities) activate varying types and numbers of peripheral nerves, thereby eliciting diverse regulatory effects in nature, scope, and duration. This effectively repairs and enhances various control functions of the urethral sphincter (50).

This study analyzed the efficacy of acupuncture combined with biofeedback electrical stimulation therapy for female stress urinary incontinence across five dimensions: clinical efficacy, frequency of leakage, leakage volume, ICI-Q-SF scores, and pelvic floor muscle strength scores. Results demonstrated that the acupuncture plus biofeedback electrical stimulation group achieved superior clinical outcomes compared to the biofeedback electrical stimulation group. The synergistic effect of acupuncture combined with biofeedback maximizes clinical efficacy. Regarding urinary leakage frequency, volume, and pelvic floor muscle strength scores, this study suggests that acupuncture combined with biofeedback may reduce urinary leakage frequency and volume, while increasing pelvic floor muscle strength scores in female patients with SUI. In acupuncture practice, the Eight Li Points (BL11-BL14) represent the most frequently utilized acupoints by clinicians for urinary disorders, reproductive system conditions, and lumbosacral pain. Research indicates that stimulating these points may regulate the discharge of the midbrain and pons (51). Furthermore, the Eight Li Points are surrounded by abundant musculature, vasculature, pelvic nerves, and the S1–S4 sacral nerves. The pelvic autonomic nerves innervate the reproductive organs and bladder, while the S1–S4 sacral nerves can elicit contractions of the detrusor muscle and sphincter. The spinal micturition center is located in the lateral horn cells of the S2–S4 spinal cord segments (52). Therefore, stimulating the Eight Liang points can improve bladder function and treat urinary incontinence. Modern medical research has also identified that the spinal nerve segments from T11 to L3 overlap with the spinal cord segments governing the bladder. The Zhongji acupoint lies within this segment, and its needling has been shown to reduce detrusor muscle contractility (53). Clinical studies indicate that acupuncture may also reduce TGF-β1 levels in pelvic tissues and serum relaxin levels. Enhancing pelvic tissue muscle strength promotes repair of pelvic tissues and bladder function, maintains urethral pressure, effectively improves urodynamics, and facilitates recovery (41). Biofeedback therapy integrates electrical stimulation with proprioceptive feedback to effectively enhance pelvic floor muscle function. Electrical stimulation directly activates the pudendal nerve, triggering neuromuscular coupling mechanisms and heightening awareness of muscle contraction (54). Concurrently, biofeedback devices convert electromyographic signals into visual or auditory feedback, allowing patients to perceive and adjust their pelvic floor muscle activity in real-time. This process strengthens sensory input pathways from muscles to the brain, enhancing awareness of muscle length, tension, and position. Combined with targeted training in vibration and positional awareness in the perineal region, it further activates the proprioceptive integration function of the motor cortex, promoting autonomous regulation of pelvic floor function (55). Consequently, the integration of acupuncture and biofeedback facilitates the repair of pelvic tissues and bladder function, significantly reducing urinary leakage frequency and volume while improving patients’ pelvic floor muscle strength scores. The ICI-Q-SF score reflects the impact of urinary incontinence on patients’ quality of life, with lower scores indicating milder symptoms and reduced daily disruption. Findings suggest that acupuncture combined with biofeedback may lower patients’ ICI-Q-SF scores. Research teams have proposed that the ‘brain-kidney-bladder’ axis represents the primary and most direct mechanism of acupuncture in treating female stress urinary incontinence. Clinical studies employing the ‘Dumai Regulation and Mental Calming’ acupuncture technique (56) selected acupoints including Baihui, Sishencong, Guanyuan, Qihai, Zhongji, Mingmen, and the Eight Liang points. With Baihui and Shenting located on the head, Guanyuan, Qihai, and Zhongji on the abdomen, and Mingmen and the Eight Li points on the lumbosacral region. This approach leverages the principle of synchronizing the brain-kidney-bladder axis to exert qi-tonifying, yang-lifting, and spirit-calming effects. It regulates the micturition center, enhances pelvic floor muscle support for the bladder and urethra, alleviates the psychological stress caused by urinary leakage in SUI patients, and reduces the impact on quality of life.

This study conducted subgroup analyses based on different acupuncture interventions. The results indicate that various acupuncture approaches significantly enhance clinical efficacy, reduce urinary leakage frequency and volume, and lower ICI-Q-SF scores. Clinical research analysis indicates that electroacupuncture stimulation at the Zhongliao and Huayang acupoints in the lumbosacral region directly stimulates the lumbosacral nerves, promoting reinnervation and strengthening of the pelvic floor muscles, thereby effectively alleviating SUI symptoms (57). Another study found that the mechanism of electroacupuncture stimulation is associated with the degradation of collagen in the anterior vaginal wall, which similarly alleviates SUI symptoms (58). Moxibustion therapy primarily exerts its therapeutic effects through thermal stimulation of the body via far-infrared radiation and the combustion of medicinal herbs within the moxa stick (59), offering fewer side effects compared to oral medications. This meta-analysis also conducted subgroup analyses based on different primary disease types. Results indicate that the acupuncture combined with biofeedback electrical stimulation group demonstrated significant therapeutic effects across various primary disease categories, with particularly pronounced efficacy for postpartum stress urinary incontinence. This is because childbirth induces mechanical compression and expansion of pelvic floor tissues, causing damage to pelvic floor muscle nerves and resulting in pelvic floor dysfunction. Acupuncture combined with biofeedback electrical stimulation therapy regulates neuromuscular excitability through electrical stimulation and acupoint stimulation, enabling passive muscle contraction and relaxation exercises to promote functional recovery (60). Regarding primary urinary incontinence, relevant studies indicate that the majority of patients present with urethral sphincter dysfunction, often stemming from congenital developmental abnormalities. Should the functional urethral length prove excessively short or bladder outlet resistance be unduly low, incontinence manifests accordingly (61). Currently, surgical intervention remains the primary treatment for primary urinary incontinence. However, acupuncture therapy, when employed as an adjunctive approach, can achieve the effects of strengthening the kidneys and reducing urine leakage, as well as warming and tonifying kidney yang. It promotes microcirculation and muscle tone in the pelvic floor, improves bladder nerve function and detrusor muscle function (21), thereby further elevating the overall therapeutic efficacy. This study also conducted subgroup analyses on the duration of acupuncture treatment. Results indicate that the acupuncture combined with biofeedback group demonstrated efficacy across varying treatment durations, with longer treatment periods yielding superior outcomes. Clinical practice confirms that the efficacy of acupuncture is closely linked to point selection, needling techniques, and treatment course design (62). A randomized controlled trial demonstrated that electroacupuncture stimulation of lumbosacral acupoints tangibly reduced urinary leakage volume, with therapeutic efficacy persisting for up to 24 weeks (63). Other research findings indicate that a combined approach utilizing reinforcing needling techniques alongside moxibustion achieves a clinical overall efficacy rate of 70–80%. Treatment cycles typically consist of 10 sessions per course, with three courses generally required to achieve optimal therapeutic outcomes (64).

According to the findings of the meta-analysis, acupuncture combined with biofeedback therapy demonstrates efficacy in treating female stress urinary incontinence. Subgroup analysis further suggests that moxibustion and electroacupuncture may be considered as the preferred acupuncture modalities for clinical management of female stress urinary incontinence. Treatment duration should be approximately 30 min per session, administered 3–5 times weekly for 8 consecutive weeks. Biofeedback therapy should last 20–30 min per session, conducted 2–3 times weekly over 8 weeks. This conclusion is for reference only, and further investigation is warranted in subsequent studies.

This study retains certain limitations. (1) The majority of included literature comprised Chinese-language publications; no foreign-language studies meeting the inclusion criteria were identified by the search cutoff date. This may have led to an overestimation of the effect size for combined therapies in this meta-analysis. Although funnel plot analysis was conducted, its statistical power remained insufficient, suggesting potential publication bias. (2) Acupuncture protocols varied considerably across included studies, with inconsistencies in needle types, point selection principles, needling techniques, and treatment frequency, thereby increasing heterogeneity between studies. (3) The outcome measure ICI-Q-SF employed in this study involves subjective reporting, potentially introducing publication bias. (4) The included studies lacked patient follow-up, with high rates of SUI recurrence observed. Different intervention methods exerted varying degrees of influence on recurrence rates. (4) This study could only validate the empirical question of whether combination therapy is more effective than monotherapy, without elucidating the underlying biological or neurophysiological mechanisms.

The combination of acupuncture and biofeedback therapy demonstrates significant efficacy in treating female stress urinary incontinence, providing valuable reference for clinical practice. However, this conclusion is influenced by factors such as the quality and quantity of included studies. The findings of this research require validation through more standardized, higher-quality clinical trials. Future studies should advance high-caliber clinical trials, standardize intervention reporting, deepen mechanistic investigations, incorporate more objective outcome measures, and establish long-term follow-up to assess treatment sustainability.

## Data Availability

The original contributions presented in the study are included in the article/Supplementary material, further inquiries can be directed to the corresponding author/s.
