# Supplementary material for: Acupuncture combined with biofeedback electrical stimulation for female stress urinary incontinence: a systematic review and meta-analysis
Source: Front Med (Lausanne). 2026 Jan 22;13:1760125. doi: 10.3389/fmed.2026.1760125 (PMC12872887; doi:10.3389/fmed.2026.1760125)
Supplement: Supplementary file 1 [file Table_1.DOCX]

Supplementary Material 1

Pubmed

Urinary Incontinence, Stress[MeSH Terms]

(((Urinary Incontinence, Stress[Title/Abstract]) OR (Urinary Stress Incontinence[Title/Abstract])) OR (Incontinence, Urinary Stress[Title/Abstract])) OR (Stress Incontinence, Urinary[Title/Abstract])

(Urinary Incontinence, Stress[MeSH Terms]) OR ((((Urinary Incontinence, Stress[Title/Abstract]) OR (Urinary Stress Incontinence[Title/Abstract])) OR (Incontinence, Urinary Stress[Title/Abstract])) OR (Stress Incontinence, Urinary[Title/Abstract]))

Acupuncture[MeSH Terms]

((((((((((((((((((((((((((((((Acupuncture[Title/Abstract]) OR (Pharmacopuncture[Title/Abstract])) OR (Acupuncture Therapy[Title/Abstract])) OR (Acupuncture Treatment[Title/Abstract])) OR (Acupuncture Treatments[Title/Abstract])) OR (Treatment, Acupuncture[Title/Abstract])) OR (Therapy, Acupuncture[Title/Abstract])) OR (Pharmacoacupuncture Treatment[Title/Abstract])) OR (Treatment, Pharmacoacupuncture[Title/Abstract])) OR (Pharmacoacupuncture Therapy[Title/Abstract])) OR (Therapy, Pharmacoacupuncture[Title/Abstract])) OR (Acupotomy[Title/Abstract])) OR (Acupotomies[Title/Abstract])) OR (Acupuncture, Ear[Title/Abstract])) OR (Acupunctures, Ear[Title/Abstract])) OR (Ear Acupunctures[Title/Abstract])) OR (Acupuncture, Auricular[Title/Abstract])) OR (Acupunctures, Auricular[Title/Abstract])) OR (Auricular Acupunctures[Title/Abstract])) OR (Auricular Acupuncture[Title/Abstract])) OR (Ear Acupuncture[Title/Abstract])) OR (Acupuncture Points[Title/Abstract])) OR (Acupuncture Point[Title/Abstract])) OR (Point, Acupuncture[Title/Abstract])) OR (Points, Acupuncture[Title/Abstract])) OR (Acupoints[Title/Abstract])) OR (Acupoint[Title/Abstract])) OR (Electroacupuncture[Title/Abstract])) OR (Moxibustion[Title/Abstract])) OR (Moxabustion[Title/Abstract])) OR (Warm acupuncture[Title/Abstract])

(Acupuncture[MeSH Terms]) OR (((((((((((((((((((((((((((((((Acupuncture[Title/Abstract]) OR (Pharmacopuncture[Title/Abstract])) OR (Acupuncture Therapy[Title/Abstract])) OR (Acupuncture Treatment[Title/Abstract])) OR (Acupuncture Treatments[Title/Abstract])) OR (Treatment, Acupuncture[Title/Abstract])) OR (Therapy, Acupuncture[Title/Abstract])) OR (Pharmacoacupuncture Treatment[Title/Abstract])) OR (Treatment, Pharmacoacupuncture[Title/Abstract])) OR (Pharmacoacupuncture Therapy[Title/Abstract])) OR (Therapy, Pharmacoacupuncture[Title/Abstract])) OR (Acupotomy[Title/Abstract])) OR (Acupotomies[Title/Abstract])) OR (Acupuncture, Ear[Title/Abstract])) OR (Acupunctures, Ear[Title/Abstract])) OR (Ear Acupunctures[Title/Abstract])) OR (Acupuncture, Auricular[Title/Abstract])) OR (Acupunctures, Auricular[Title/Abstract])) OR (Auricular Acupunctures[Title/Abstract])) OR (Auricular Acupuncture[Title/Abstract])) OR (Ear Acupuncture[Title/Abstract])) OR (Acupuncture Points[Title/Abstract])) OR (Acupuncture Point[Title/Abstract])) OR (Point, Acupuncture[Title/Abstract])) OR (Points, Acupuncture[Title/Abstract])) OR (Acupoints[Title/Abstract])) OR (Acupoint[Title/Abstract])) OR (Electroacupuncture[Title/Abstract])) OR (Moxibustion[Title/Abstract])) OR (Moxabustion[Title/Abstract])) OR (Warm acupuncture[Title/Abstract]))

((Urinary Incontinence, Stress[MeSH Terms]) OR ((((Urinary Incontinence, Stress[Title/Abstract]) OR (Urinary Stress Incontinence[Title/Abstract])) OR (Incontinence, Urinary Stress[Title/Abstract])) OR (Stress Incontinence, Urinary[Title/Abstract]))) AND ((Acupuncture[MeSH Terms]) OR (((((((((((((((((((((((((((((((Acupuncture[Title/Abstract]) OR (Pharmacopuncture[Title/Abstract])) OR (Acupuncture Therapy[Title/Abstract])) OR (Acupuncture Treatment[Title/Abstract])) OR (Acupuncture Treatments[Title/Abstract])) OR (Treatment, Acupuncture[Title/Abstract])) OR (Therapy, Acupuncture[Title/Abstract])) OR (Pharmacoacupuncture Treatment[Title/Abstract])) OR (Treatment, Pharmacoacupuncture[Title/Abstract])) OR (Pharmacoacupuncture Therapy[Title/Abstract])) OR (Therapy, Pharmacoacupuncture[Title/Abstract])) OR (Acupotomy[Title/Abstract])) OR (Acupotomies[Title/Abstract])) OR (Acupuncture, Ear[Title/Abstract])) OR (Acupunctures, Ear[Title/Abstract])) OR (Ear Acupunctures[Title/Abstract])) OR (Acupuncture, Auricular[Title/Abstract])) OR (Acupunctures, Auricular[Title/Abstract])) OR (Auricular Acupunctures[Title/Abstract])) OR (Auricular Acupuncture[Title/Abstract])) OR (Ear Acupuncture[Title/Abstract])) OR (Acupuncture Points[Title/Abstract])) OR (Acupuncture Point[Title/Abstract])) OR (Point, Acupuncture[Title/Abstract])) OR (Points, Acupuncture[Title/Abstract])) OR (Acupoints[Title/Abstract])) OR (Acupoint[Title/Abstract])) OR (Electroacupuncture[Title/Abstract])) OR (Moxibustion[Title/Abstract])) OR (Moxabustion[Title/Abstract])) OR (Warm acupuncture[Title/Abstract])))

Embase

#4 'stress incontinence'/exp

#5 'urinary stress incontinence':ab,ti

#6 'incontinence, urinary stress':ab,ti

#7 'stress incontinence, urinary':ab,ti

#8 'urinary incontinence, stress':ab,ti

#9 #4 OR #5 OR #6 OR #7 OR #8

#10 'acupuncture'/exp

#11 'acupuncture':ab,ti

#12 'pharmacopuncture':ab,ti

#13 'acupuncture therapy':ab,ti

#14 'acupuncture treatment':ab,ti

#15 'acupuncture treatments':ab,ti

#16 'treatment, acupuncture':ab,ti

#17 'therapy, acupuncture':ab,ti

#18 'pharmacoacupuncture treatment':ab,ti

#19 'treatment, pharmacoacupuncture':ab,ti

#20 'pharmacoacupuncture therapy':ab,ti

#21 'therapy, pharmacoacupuncture':ab,ti

#22 'acupotomy':ab,ti

#23 'acupotomies':ab,ti

#24 'acupuncture, ear':ab,ti

#25 'acupunctures, ear':ab,ti

#26 'ear acupunctures':ab,ti

#27 'acupuncture, auricular':ab,ti

#28 'acupunctures, auricular':ab,ti

#29 'auricular acupunctures':ab,ti

#30 'auricular acupuncture':ab,ti

#31 'ear acupuncture':ab,ti

#32 'acupuncture points':ab,ti

#33 'acupuncture point':ab,ti

#34 'point, acupuncture':ab,ti

#35 'points, acupuncture':ab,ti

#36 'acupoints':ab,ti

#37 'acupoint':ab,ti

#38 'electroacupuncture':ab,ti

#39 'moxibustion':ab,ti

#40 'moxabustion':ab,ti

#41 'warm acupuncture':ab,ti

#42 #10 OR #11 OR #12 OR #13 OR #14 OR #15 OR #16 OR #17 OR #18 OR #19 OR #20 OR #21 OR #22 OR #23 OR #24 OR #25 OR #26 OR #27 OR #28 OR #29 OR #30 OR #31 OR #32 OR #33 OR #34 OR #35 OR #36 OR #37 OR #38 OR #39 OR #40 OR #41

#43 #9 AND #42

Web of Science

TS=(Urinary Incontinence, Stress) OR TS=(Urinary Stress Incontinence) OR TS=(Incontinence, Urinary Stress) OR TS=(Stress Incontinence, Urinary) and Preprint Citation Index (Exclude – Database)

TS=(Acupuncture) OR TS=(Pharmacopuncture) OR TS=(Acupuncture Therapy) OR TS=(Acupuncture Treatment) OR TS=(Acupuncture Treatments) OR TS=(Treatment, Acupuncture) OR TS=(Therapy, Acupuncture) OR TS=(Pharmacoacupuncture Treatment) OR TS=(Treatment, Pharmacoacupuncture) OR TS=(Pharmacoacupuncture Therapy) OR TS=(Therapy, Pharmacoacupuncture) OR TS=(Acupotomy) OR TS=(Acupotomies) OR TS=(Acupuncture, Ear) OR TS=(Acupunctures, Ear) OR TS=(Ear Acupunctures) OR TS=(Acupuncture, Auricular) OR TS=(Acupunctures, Auricular) OR TS=(Auricular Acupunctures) OR TS=(Auricular Acupuncture) OR TS=(Ear Acupuncture) OR TS=(Acupuncture Points) OR TS=(Acupuncture Point) OR TS=(Point, Acupuncture) OR TS=(Points, Acupuncture) OR TS=(Acupoints) OR TS=(Acupoint) OR TS=(Electroacupuncture) OR TS=(Moxibustion) OR TS=(Moxabustion) OR TS=(Warm acupuncture) and Preprint Citation Index (Exclude – Database)

#2 AND #1 and Preprint Citation Index (Exclude – Database)

#1 MeSH descriptor: [Urinary Incontinence, Stress] explode all trees 1573

#2 (Urinary Incontinence, Stress):ti,ab,kw OR (Urinary Stress Incontinence):ti,ab,kw OR (Incontinence, Urinary Stress):ti,ab,kw OR (Stress Incontinence, Urinary):ti,ab,kw 3737

#3 #1 or #2 3737

#4 MeSH descriptor: [Acupuncture] explode all trees 216

#5 (Acupuncture):ti,ab,kw OR (Pharmacopuncture):ti,ab,kw OR (Acupuncture Therapy):ti,ab,kw OR (Acupuncture Treatment):ti,ab,kw OR (Acupuncture Treatments):ti,ab,kw 21451

#6 (Treatment, Acupuncture):ti,ab,kw OR (Therapy, Acupuncture):ti,ab,kw OR (Pharmacoacupuncture Treatment):ti,ab,kw OR (Treatment, Pharmacoacupuncture):ti,ab,kw OR (Pharmacoacupuncture Therapy):ti,ab,kw 17186

#7 (Therapy, Pharmacoacupuncture):ti,ab,kw OR (Acupotomy):ti,ab,kw OR (Acupotomies):ti,ab,kw OR (Acupuncture, Ear):ti,ab,kw OR (Acupunctures, Ear):ti,ab,kw 1109

#8 (Ear Acupunctures):ti,ab,kw OR (Acupuncture, Auricular):ti,ab,kw OR (Acupunctures, Auricular):ti,ab,kw OR (Auricular Acupunctures):ti,ab,kw OR (Auricular Acupuncture):ti,ab,kw 1076

#9 (Ear Acupuncture):ti,ab,kw OR (Acupuncture Points):ti,ab,kw OR (Acupuncture Point):ti,ab,kw OR (Point, Acupuncture):ti,ab,kw OR (Points, Acupuncture):ti,ab,kw 8697

#10 (Acupoints):ti,ab,kw OR (Acupoint):ti,ab,kw OR (Electroacupuncture):ti,ab,kw OR (Moxibustion):ti,ab,kw OR (Moxabustion):ti,ab,kw 11527

#11 (Warm acupuncture):ti,ab,kw 302

#12 #4 or #5 or #6 or #7 or #8 or #9 or #10 or #11 25958

#13 #3 and #12 107


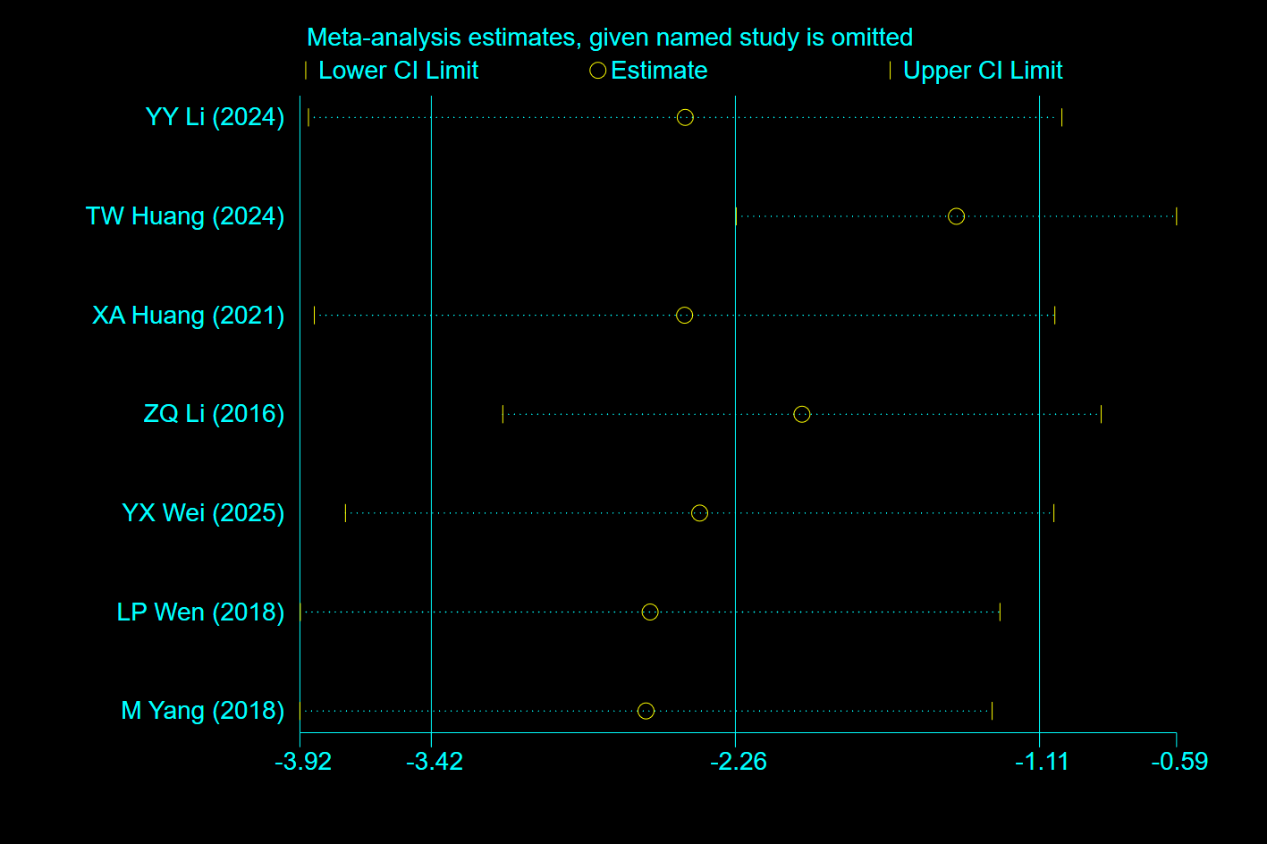


Supplementary Material Figure 1 Sensitivity analysis of the number of urine leaks


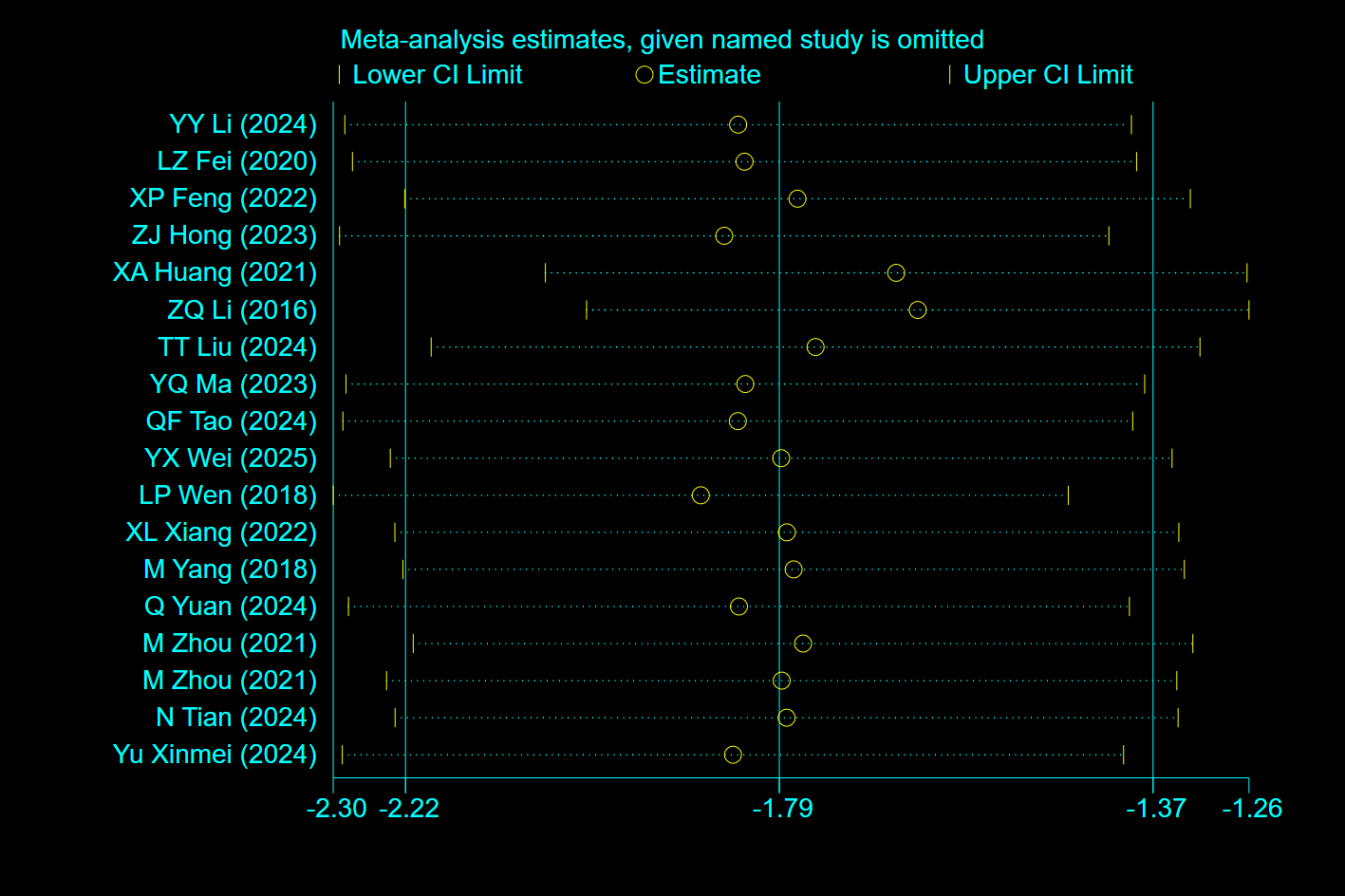


Supplementary Material Figure 2 Urine leakage sensitivity analysis


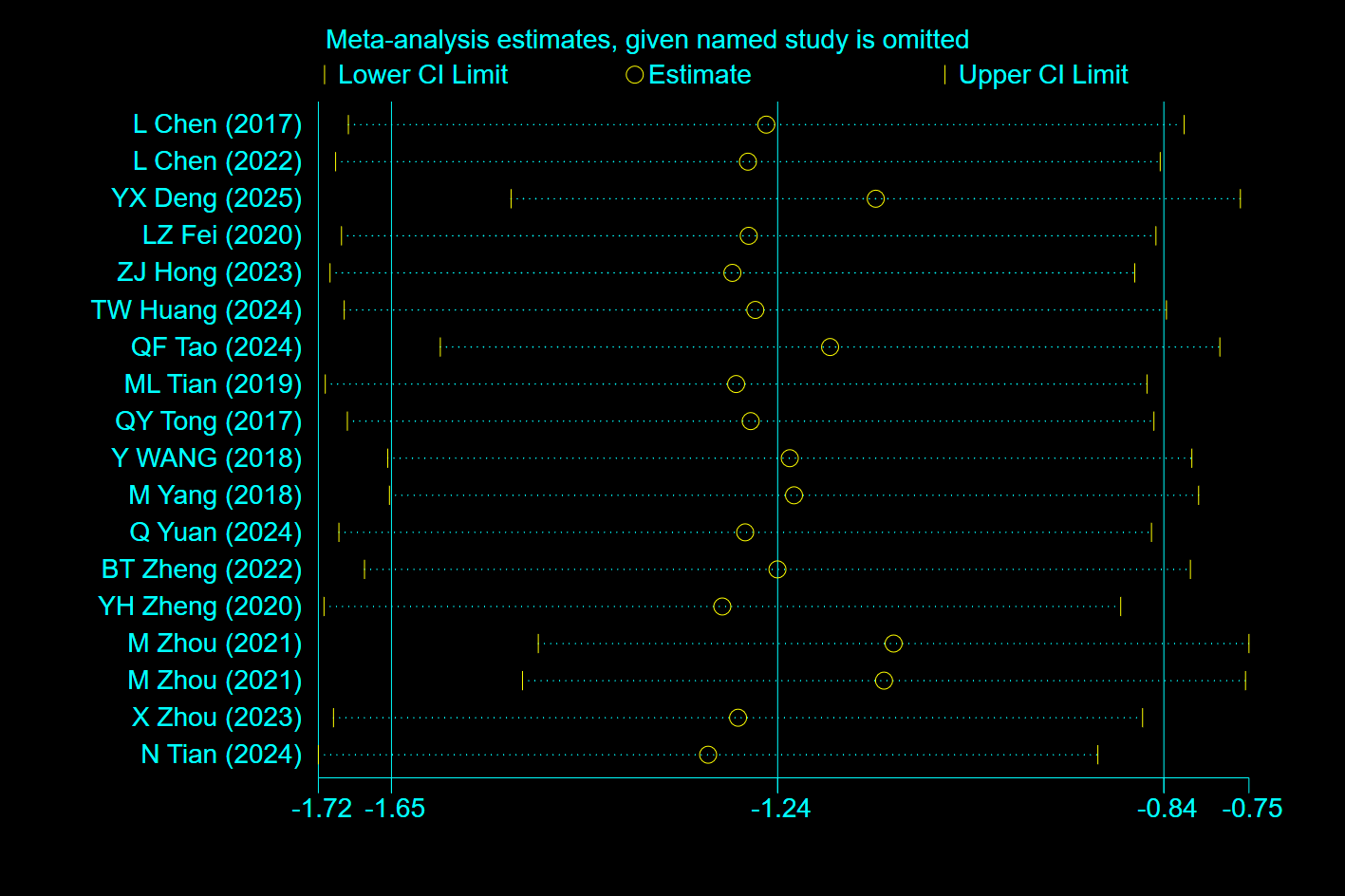


Supplementary Material Figure 3 Sensitivity analysis of ICI-Q-SF scores


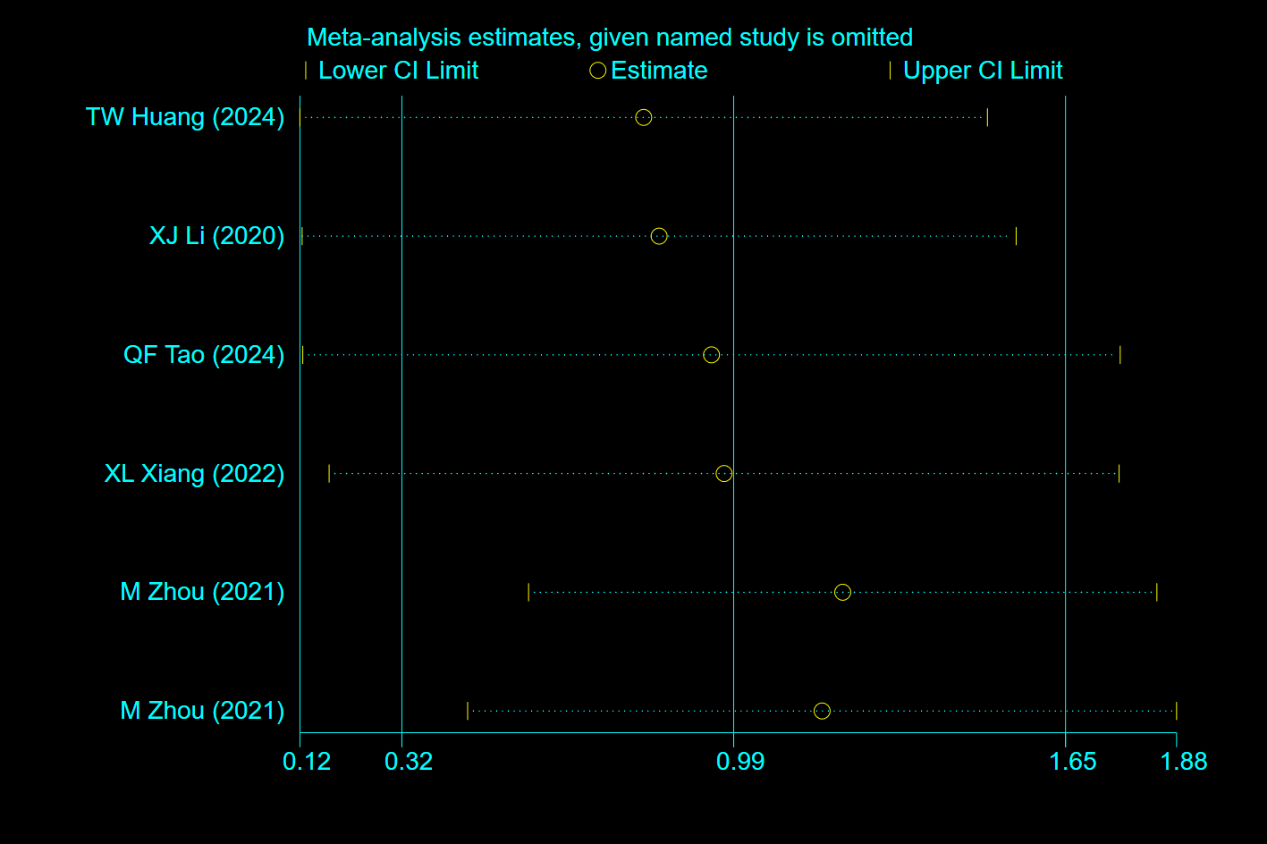


Supplementary Material Figure 4 Sensitivity analysis of pelvic floor muscle strength scores


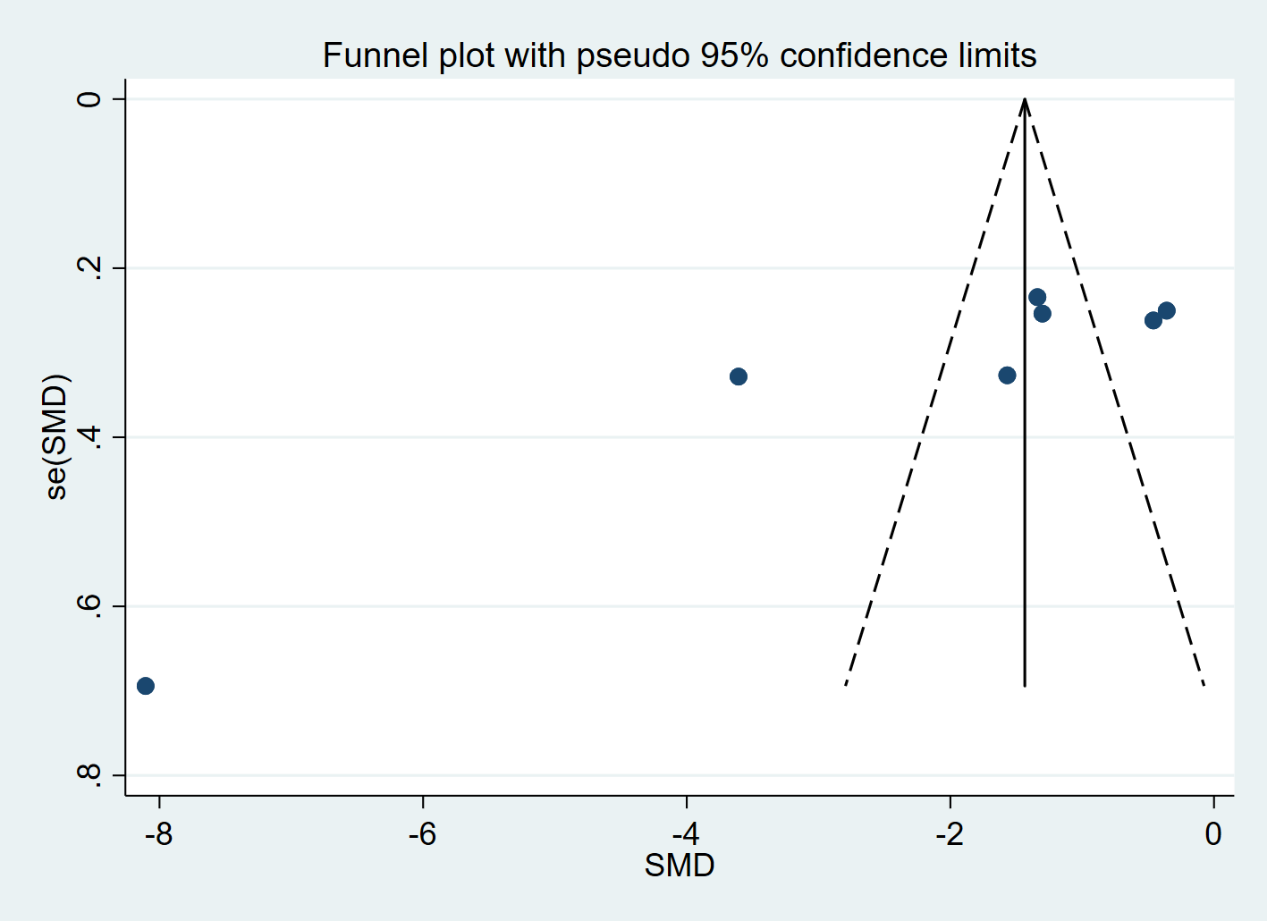


Supplementary Material Figure 5 Funnel plot of the number of urine leaks


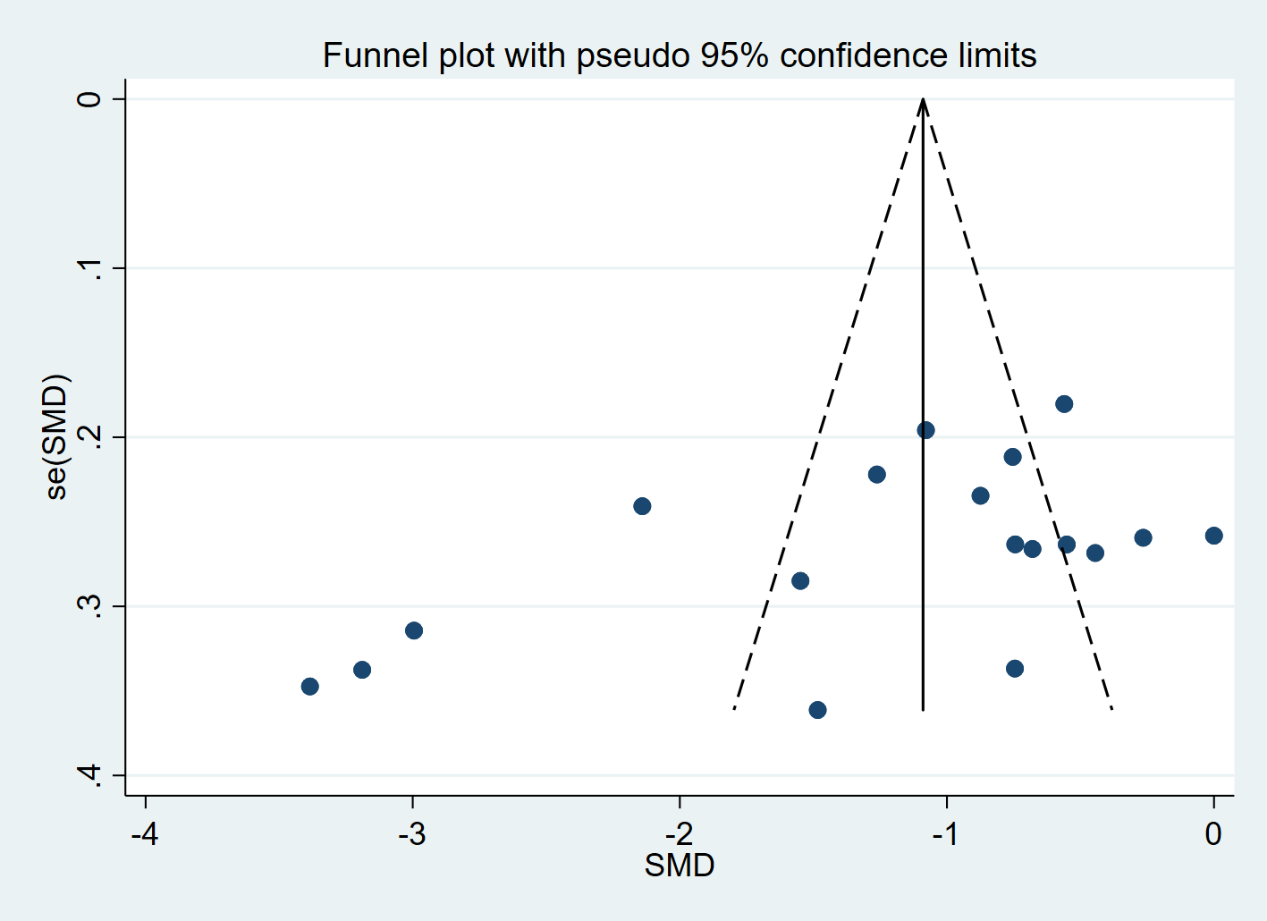


Supplementary Material Figure 6 Funnel plot of ICI-Q-SF scores


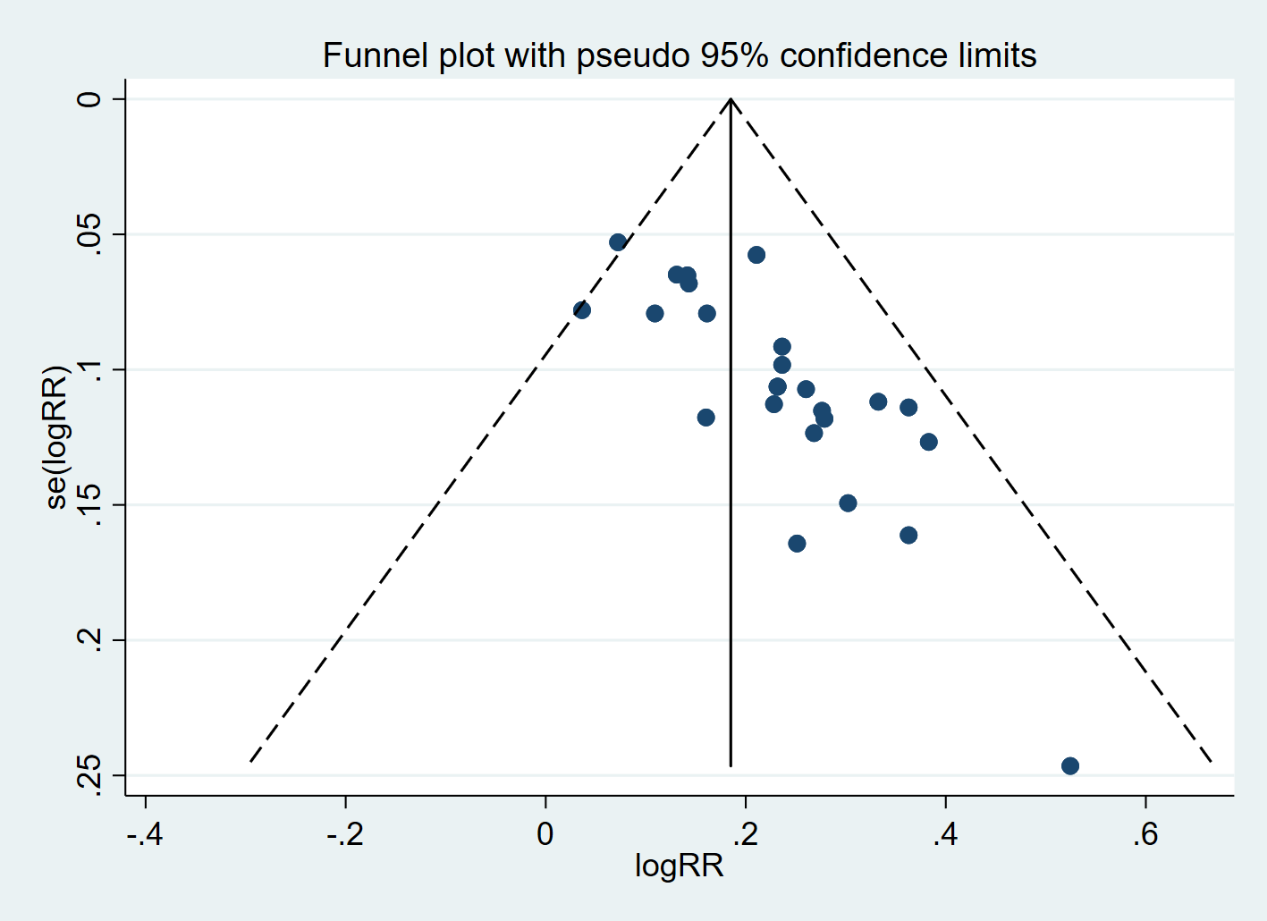


Supplementary Material Figure 7 Funnel plot of clinical efficacy


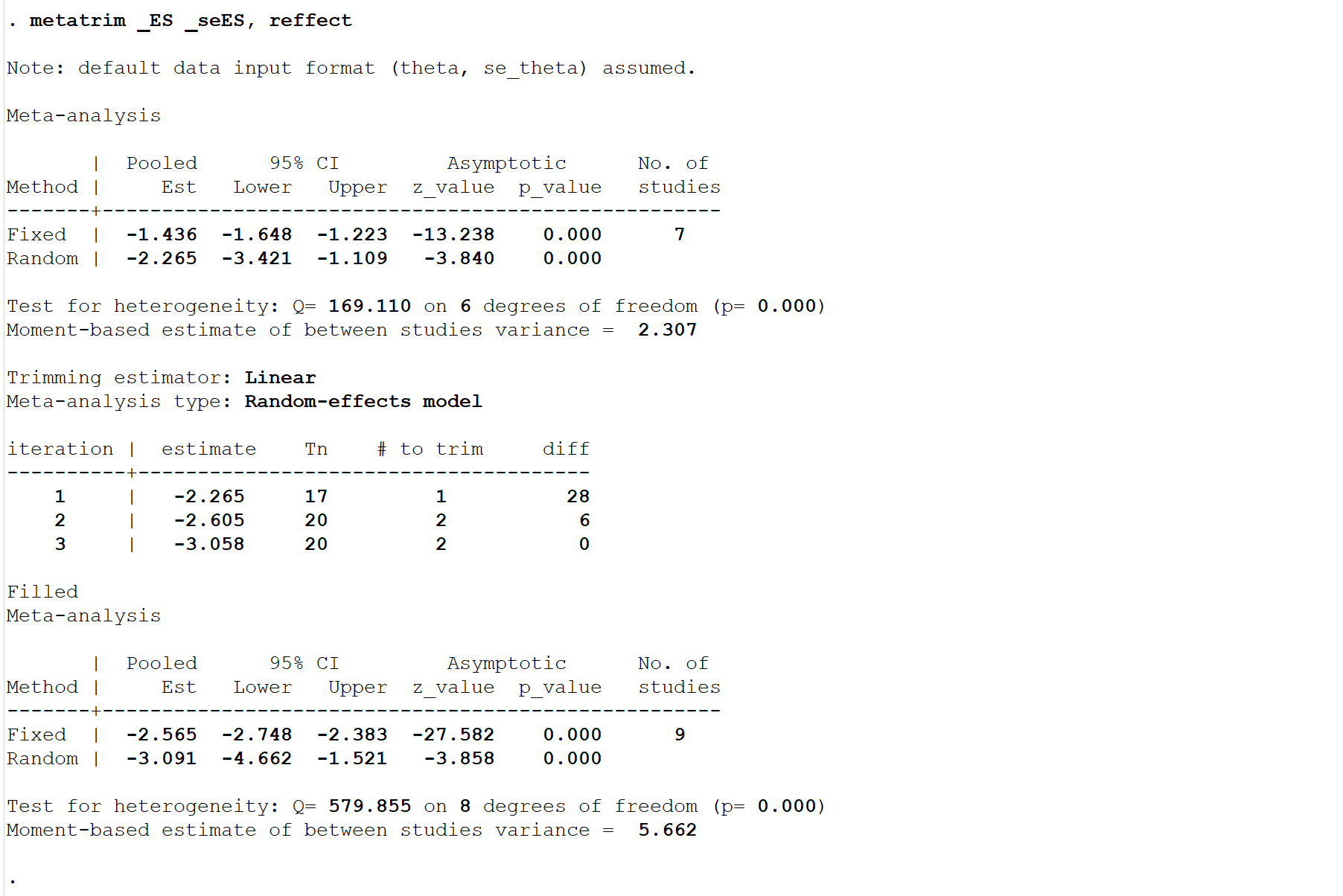


Supplementary material Figure 8 Clipping and filling method for the number of urine leakage


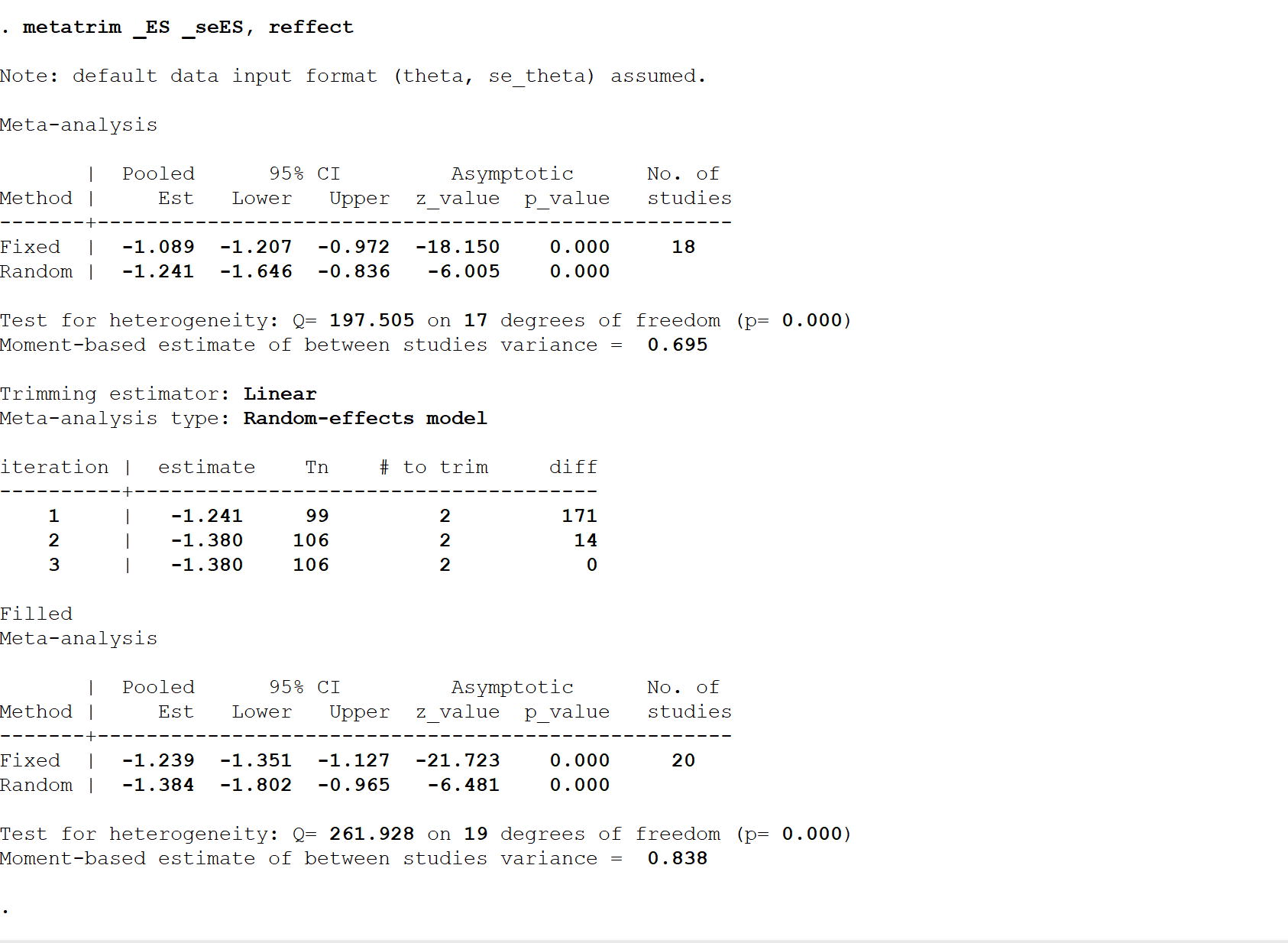


Supplementary material Figure 9 clip-fill method for ICI-Q-SF scoring


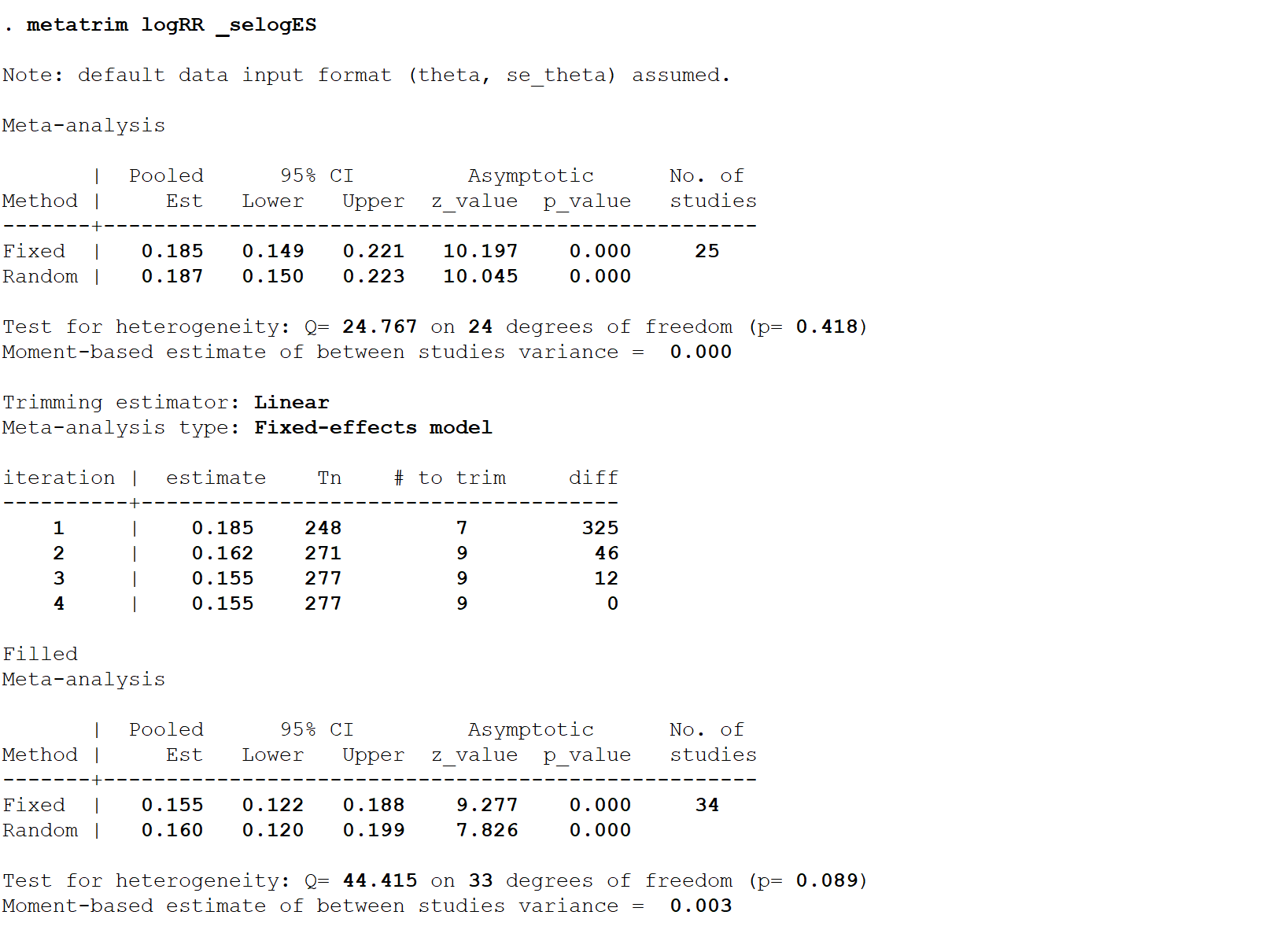


Supplementary Material Figure 10 Clinical efficacy clipping and filling method


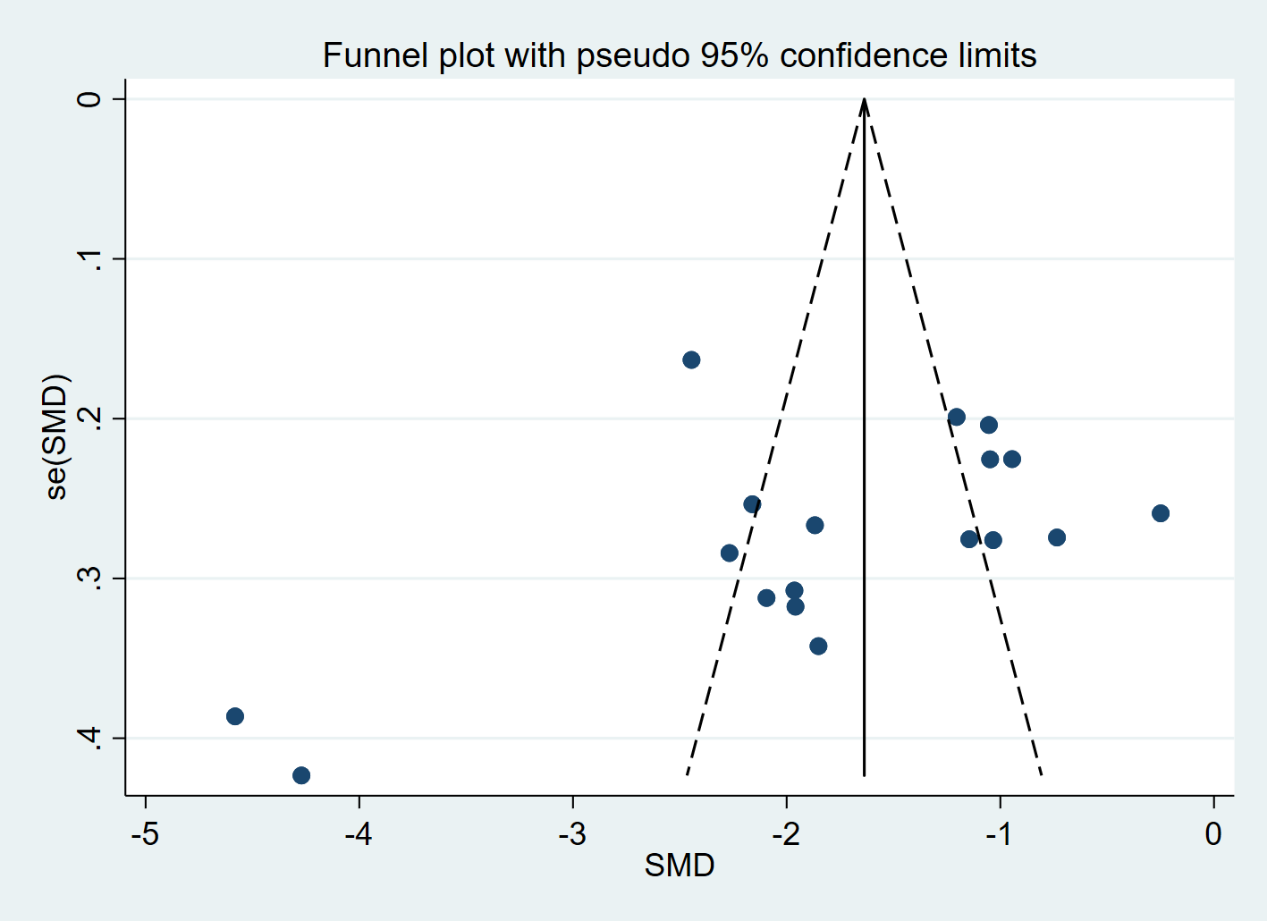


Supplementary Material Figure 11 Funnel plot of urine leakage volume


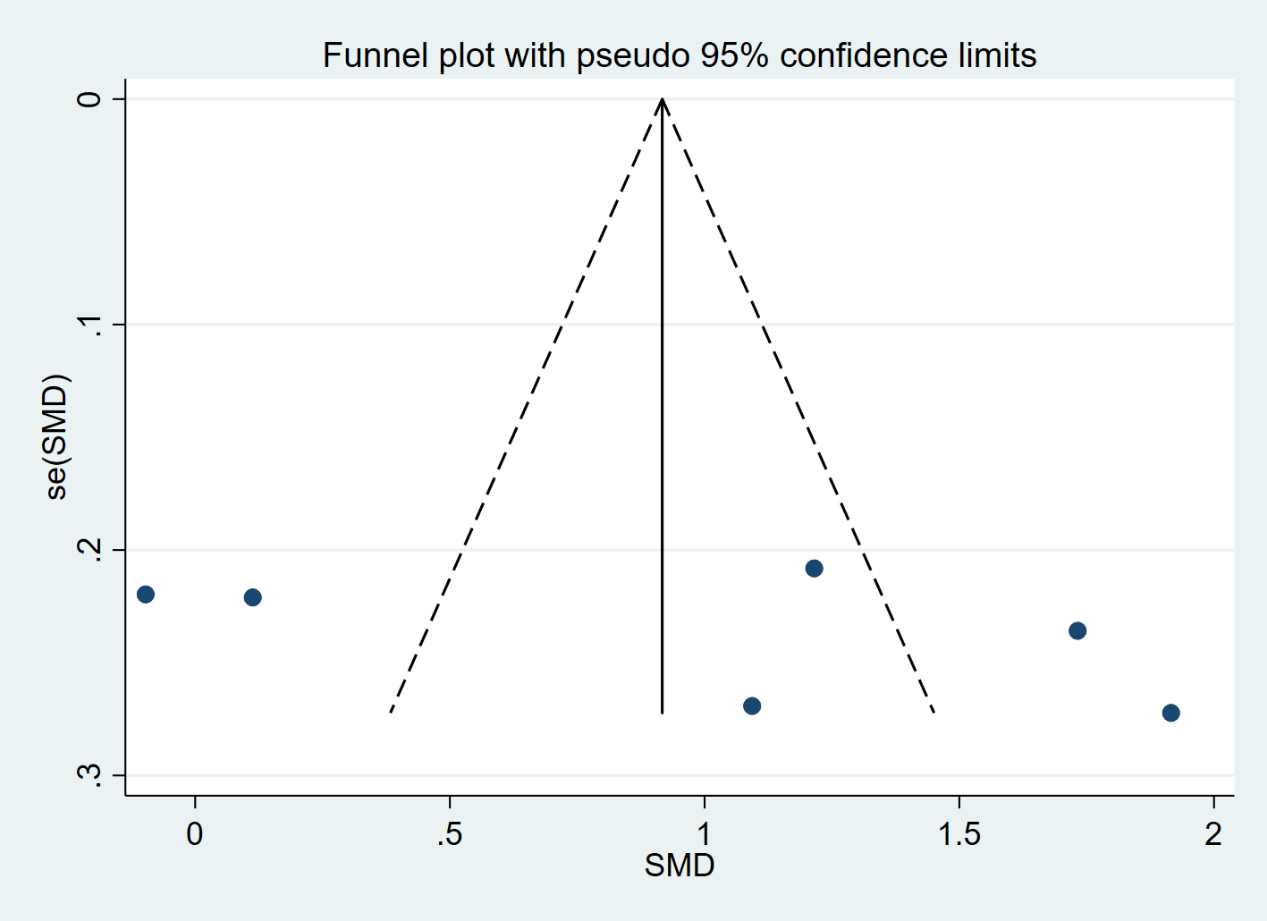


Supplementary Material Figure 12 Funnel plot of pelvic floor muscle strength scores
